# Hyaluronan Is Crucial for Stem Cell Differentiation into Smooth Muscle Lineage

**DOI:** 10.1002/stem.2328

**Published:** 2016-03-04

**Authors:** Russell M.L. Simpson, Xuechong Hong, Mei Mei Wong, Eirini Karamariti, Shirin Issa Bhaloo, Derek Warren, Wei Kong, Yanhua Hu, Qingbo Xu

**Affiliations:** ^1^ Cardiovascular Division, BHF Centre for Vascular Regeneration, King's College LondonLondonUnited Kingdom; ^2^ Department of Physiology and Pathophysiology, School of Basic Medical Sciences, Key Laboratory of Molecular Cardiovascular Science, Ministry of Education, Peking UniversityBeijingChina

**Keywords:** Hyaluronan, Stem cells, Smooth muscle cells, Vasculogenesis, Neointima

## Abstract

Deciphering the extracellular signals that regulate SMC differentiation from stem cells is vital to further our understanding of the pathogenesis of vascular disease and for development of cell‐based therapies and tissue engineering. Hyaluronan (HA) has emerged as an important component of the stem cell niche, however its role during stem cell differentiation is a complicated and inadequately defined process. This study aimed to investigate the role of HA in embryonic stem cell (ESC) differentiation toward a SMC lineage. ESCs were seeded on collagen‐IV in differentiation medium to generate ESC‐derived SMCs (esSMCs). Differentiation coincided with increased HA synthase (HAS) 2 expression, accumulation of extracellular HA and its assembly into pericellular matrices. Inhibition of HA synthesis by 4‐methylumbelliferone (4MU), removal of the HA coat by hyaluronidase (HYAL) or HAS2 knockdown led to abrogation of SMC gene expression. HA activates ERK1/2 and suppresses EGFR signaling pathways via its principle receptor, CD44. EGFR inactivation coincided with increased binding to CD44, which was further augmented by addition of high molecular weight (HMW)‐HA either exogenously or via HAS2 overexpression through adenoviral gene transfer. HMW‐HA‐stimulated esSMCs displayed a functional role in vascular tissue engineering ex vivo, vasculogenesis in a matrigel plug model and SMC accumulation in neointimal lesions of vein grafts in mice. These findings demonstrate that HAS2‐induced HA synthesis and organization drives ESC‐SMC differentiation. Thus, remodeling of the HA microenvironment is a critical step in directing stem cell differentiation toward a vascular lineage, highlighting HA as a potential target for treatment of vascular diseases. Stem Cells *2016;34:1225–1238*

**Significance Statement**

Stem cell differentiation toward a smooth muscle cell (SMC) lineage plays a significant role in the pathogenesis of vascular disease but can also offer an alternative cell source for the development of stem cell‐based tissue engineering strategies. This body of work demonstrates that synthesis and pericellular organization of hyaluronan, an important stem cell niche component, provide the extracellular signaling cues necessary to direct stem cell‐SMC differentiation. The possibility of controlling stem cell fate by manipulating hyaluronan (HA) within the niche directly, or by targeting the pathways which regulate HA homeostasis, may provide significant improvement for clinical therapy in vascular diseases.

## Introduction

Embryonic stem cells (ESCs) 1, 2 are pluripotent derivatives of the inner cell mass of blastocysts. Owing to their dual ability for self‐renewal and differentiation into vascular lineages, ESCs serve as a promising source of smooth muscle cells (SMCs) for vascular tissue engineering, angiogenesis and vasculogenesis. Elucidating the molecular mechanisms of SMC differentiation from stem cells will thus be decisive in developing new cell‐based treatments for vascular disease. One emerging theme is that stem cell plasticity and fate is dependent on their ability to actively respond to extracellular cues from the surrounding microenvironment or “niche” where stem cells reside. We and others have demonstrated that ESCs can differentiate into SMCs in response to major functional niche components including growth factors for example TGF‐β 3 and PDGF‐BB 4, contact with supporting niche cells 5, mechanical stress 6 and signals from the extracellular matrix (ECM) 7. More recently, hyaluronan has been identified as a major matrix constituent of the stem cell niche 8, 9, 10, 11, 12, 13.

Hyaluronan (hyaluronic acid; HA) is a ubiquitous, hydrophilic and nonsulfated glycosaminoglycan composed of repeating disaccharide units of d‐glucoronic acid and *N*‐acetylglucosamine. It is synthesized at the plasma membrane via three membrane bound HA synthase (HAS) isoforms (HAS1, HAS2, HAS3) 14, 15 and carries out innumerous biological functions that are essential for, embryogenesis 9, 16, differentiation 17, 18, 19, 20, migration 21, 22, 23, proliferation 20, 24, 25 and intercellular communication 26 in a variety of cell types. The wide range of biological actions of HA derives from its versatile biosynthesis and organization, which is regulated in a flexible manner via the three HAS isoforms which differ in intrinsic enzymic properties, HA elongation size, rate of biosynthesis and expression patterns 27, 28, 29, 30, 31, 32. Accumulating evidence demonstrates that HA plays a role in many facets of stem cell biology 33. Enhanced HA synthesis during embryoid body differentiation by ESCs is associated with epithelial‐mesenchymal transition 16. Furthermore, differentiation of ESCs toward hematopoietic cells is reliant on HA synthesis 34. More recently, HA synthesis and assembly into a pericellular coat was shown to provide a protective niche for maintaining “stemness” in mesenchymal stem cells 8, a property which has since been exploited in the development of HA‐based hydrogel scaffolds for tissue engineering/vascular regeneration 35, 36, 37, 38.

However, the role of HA in the regulation of ESC‐SMC differentiation remains unclear. In the current study, we provide novel evidence that ESC‐SMC differentiation is dependent on extracellular signals received via remodeling of the HA microenvironment. Critically, impairment of HA synthesis and pericellular organization averts ESC‐SMC differentiation. We demonstrate in vivo that in an artificial HA microenvironment, ESC‐derived SMCs (esSMCs) exhibit superior angiogenic and vasculogenic potential. Furthermore they acquire an enhanced migratory SMC phenotype, which promotes neointima formation in a vessel graft model. Taken together, the prospect of regulating stem cell fate by HA manipulation may provide new regenerative therapies for the treatment of vascular disease.

## Materials and Methods

Detailed Methods are included in the Supporting Information.

### Generation of esSMCs

esSMCs were generated as previously described 4. Briefly, mouse ESCs were seeded on collagen IV (5 μg/ml) and differentiated toward SMCs in differentiation media (DM) for up to 8 days, after which they were harvested and further analyzed.

### HA Measurement

Extracellular HA secretion was evaluated using a HA binding protein (HABP)‐based ELISA kit. Pericellular and intracellular HA was visualized using biotinylated HABP (2 μg/ml) staining followed by incubation with Streptavidin.

### HA Inhibition/Stimulation

To investigate the effects of HA deprivation on SMC differentiation, esSMCs were treated with 4‐methylumbelliferone (4MU) (0.5 mmol/l) to inhibit HA synthesis, treated with hyaluronidase (HYAL) (200 μg/ml) to disrupt HA coat formation or transfected with a specific siRNA targeting HAS2. esSMCs were cultured in low molecular weight‐ (LMW‐) or high molecular weight‐ (HMW‐) HA (200 μg/ml) or transfected with a HAS2‐HA adenovirus to investigate the effects of HA stimulation.

### Ex Vivo Bioreactor

To study esSMCs migrating across the vessel wall in response to HA stimulation, ESCs were seeded on decellularized vessels connected to a constructed bio‐rector (an ex vivo circulation system which is driven by a pump). After 8 days of −/ + HMW‐HA stimulation, tissue engineered vessels were harvested and analyzed.

### In Vivo Angiogenesis Assay

ESC‐derived endothelial cells (esECs) were mixed equally with −/ + HA stimulated esSMCs (0.5 × 10^6^) in Matrigel and subcutaneously injected into TIE2‐LacZ transgenic mice. One week later the plugs were harvested and stained for SMC/EC markers to assess the extent of vessel formation and interaction.

### Vein Graft

The procedure used for vein grafts was similar to that described previously 39. Briefly, the vena cava vein was harvested from an isogenic donor and grafted between the two ends of the carotid artery in ApoE^−/−^ mice. −/ + HMW‐HA stimulated esSMCs (5 × 10^5^) were applied to the adventitial side to envelope the graft. The graft was harvested after 2 weeks and analyzed for neointimal formation.

## Results

### Extracellular HA Synthesis is Upregulated During ESC‐SMC Differentiation

**Figure 1:**
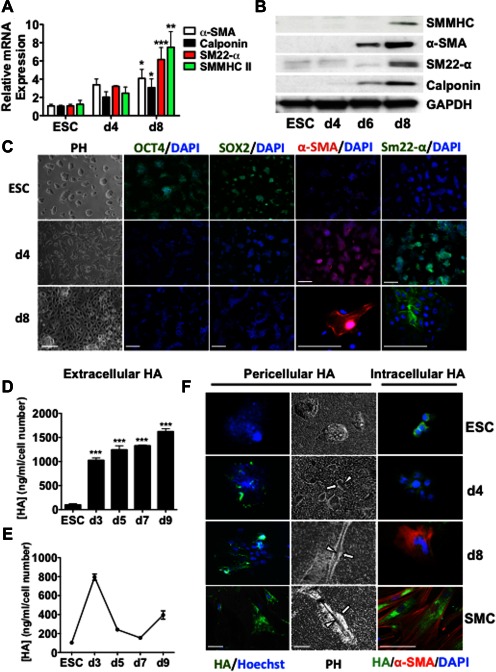
ESC‐SMC differentiation coincides with changes in HA synthesis and organization. ESCs were seeded on collagen IV‐coated plates (5 μg/ml) and cultured in differentiation medium for up to 8 days. **(A)**: RT‐qPCR analysis demonstrated augmented levels of the SMC markers, α‐SMA, calponin, sm22‐α and SMMHC over time with significant induction at day 8 (mean ± SEM, *n* = 3). **(B)**: Western blot of SMMHC, calponin, sm22‐α and α‐SMA in undifferentiated ESCs (ESC) and differentiated ESCs (d4,6,8). **(C)**: PHs show round colony‐like ESCs take on a SMC‐like morphology under differentiation conditions (d4,8). Immunofluorescence staining for pluripotency markers, OCT4 and SOX2 and SMC markers α‐SMA, calponin and sm22‐α in undifferentiated ESCs and day 4 and 8 of differentiation. A quantitative HA‐binding protein‐based kit was used to measure total **(D)** and de novo **(E)**: HA secreted in medium conditioned by undifferentiated ESCs and differentiated ESCs (d2,4,6,8) (mean ± SEM, *n* = 3). **(F)**: Pericellular HA was visualized by immunofluorescence staining with a biotinylated HA‐binding probe to localize HA (green) in live cells (left panel) or by using a particle‐exclusion assay (right panel), HA‐dependent pericellular coats are apparent as a clear zone between cells and formalized horse erythrocytes, arrows indicate the cell body; arrowheads show the extent of the pericellular matrix. ESCs differentiated toward a SMC phenotype for 4 and 8 days present increased pericellular HA coat assembly, comparable to mature SMCs (SMC). Intracellular HA was visualized by dual immunofluorescence staining with a biotinylated HA‐binding probe to localize HA (green) and α‐SMA (red). Differentiation of ESCs toward SMC phenotype is characterized by loss of intracellular HA deposition whereas mature SMCs (SMC) display significant intracellular HA staining. Bars, 100 μm. Images and blots shown are representative of at least three separate experiments, graphs are shown as mean ± SEM of at least three independent experiments. Analyses based on one‐way ANOVA, followed by Bonferroni's Multiple Comparison Test. *, *p* < .05; **, *p* < .01; ***, *p* < .001 versus ESC. Abbreviations: ESC, embryonic stem cell; HA, hyaluronan; PHs, phase contrast images; SMC, smooth muscle cell; RT‐qPCR, quantitative reverse transcription polymerase chain reaction.

Undifferentiated mouse ES cells were seeded onto collagen‐IV‐coated flasks and cultured in DM in the absence of leukemia inhibitory factor for 1–8 days to induce SMC differentiation, as previously described 4. We found that a panel of SMC‐specific genes including α‐sma, calponin, sm22‐α and smmhc were significantly upregulated at day 8 of differentiation, as confirmed by RTq‐PCR (Fig. 1A) and Western blot (Fig. 1B). Immunofluorescence staining (Fig. 1C) also showed that by day 8 of differentiation, ESCs were strongly positive for the expression of α‐sma, and sm22‐α, confirming that ESCs had been efficiently differentiated toward a smooth muscle phenotype. Conversely, as expected, there was no apparent staining for the pluripotency markers Oct4 and Sox2, which were observed in undifferentiated ESCs. Furthermore, phase contrast images showed that by day 8 of differentiation, the spherical‐like colony shape, characteristic of ESCs, had taken on a SMC‐like morphology (Fig. 1C). We defined these cells as esSMCs. Importantly, esSMCs displayed SMC marker expression comparable with mature SMCs (Supporting Information Fig. 1A). Furthermore, esSMCs showed significant collagen gel contractility compared to undifferentiated ESCs (Supporting Information Fig. 1B) and were able to contract in response to KCl stimulation (Supporting Information Online Video 1).

Very little is known about the production of HA by stem cells, in particular during their differentiation. To address this, the amount of extracellular HA secreted into the conditioned medium was collected throughout ESC‐SMC differentiation and measured using an ELISA‐type assay. Undifferentiated ESCs synthesized very low levels of extracellular HA but within 72 hours of differentiation, total HA production was significantly upregulated (∼7.8‐fold) and sustained over the course of differentiation (Fig. 1D). Analysis of de novo production revealed that the majority of HA synthesis occurs at day 3 of differentiation and dramatically drops by day 5 and day 7 (Fig. 1E).

To further understand the role of HA in ESC‐SMC differentiation, supernatants were taken at different days (0, 3, 5 and 7) of differentiation in the absence or presence of 4MU, a small molecule inhibitor of HA synthesis, which reduces the available intracellular pool of UDP‐glucuronic acid 40. The conditioned media collected was then used to directly culture ESCs for 48 hours. RTq‐PCR analysis demonstrated that conditioned medium from day 3 supernatant was able to support significant SMC marker gene induction. This induction was ablated when culturing ESCs with 4MU‐treated media, indicating this effect was specific to HA accumulation (Supporting Information Fig. 2). As well as extracellular HA content, we also assessed pericellular and intracellular HA (Fig. 1F). To monitor the extent of a pericellular HA, live ESCs were incubated with a biotinylated HA‐binding probe and observed in undifferentiated conditions and at day 4 and 8 of differentiation. Pericellular HA staining was absent in undifferentiated ESCs but was increasingly prominent with differentiation (d4‐8). Notably, esSMCs demonstrated significant HA staining comparable to that of mature SMCs (Fig. 1F, pericellular HA, left panel). A particle exclusion assay was also used to verify these findings. In this assay erythrocytes are excluded from the cell membrane by the large size and negative charge of any pericellular HA. This is observed under the microscope as a zone of erythrocyte exclusion surrounding the cells. Whilst undifferentiated ESCs failed to assemble a coat, day 4 and day 8 differentiated ESCs displayed a notable halo‐like HA coat, which was comparable to that of mature SMCs (Fig. 1F, pericellular HA, right panel). Investigation of intracellular HA in undifferentiated ESCs demonstrated intense HA staining which appeared to diminish with SMC differentiation. esSMCs (d8) stained positive for the SMC marker α‐sma with barely detectable HA expression. Conversely mature SMCs exhibited significant intracellular HA staining (Fig. 1F, intracellular HA). To investigate the relationship between intracellular HA and SMC differentiation further, day 3 differentiating ESCs were fixed permeabilized and stained for HA and sm22‐α. An extensive outgrowth of sm22‐α positive cells were observed which had migrated and differentiated from the highly HA‐stained central cellular core (Supporting Information Fig. 3).

### HA Synthesis and Pericellular Retention Drives ESC‐SMC Differentiation

**Figure 2:**
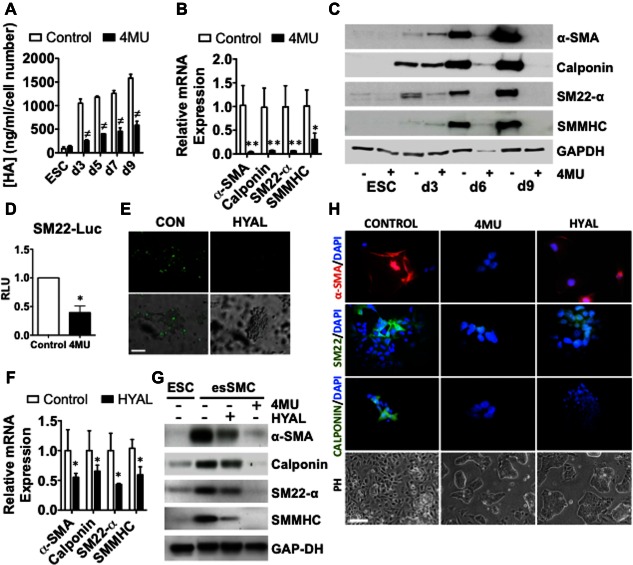
HA synthesis and pericellular organization drives ESC‐smooth muscle cell (SMC) differentiation. ESCs were seeded on collagen IV‐coated plates (5 μg/ml) and cultured in differentiation medium for up to 8 days with or without treatment with HA synthesis inhibitor 4MU (0.5 mmol/L) or HYAL (200 μg/ml) to digest the HA coat. **(A)**: Quantification by a HA‐binding protein‐based ELISA confirms that in the presence of 4MU, accumulation of total extracellular HA synthesis is ablated during the course of ESC‐SMC differentiation. **(B)**: RT‐qPCR analysis demonstrates that α‐SMA, calponin, sm22‐α and SMMHC expression in esSMCs is significantly attenuated in the presence of 4MU. **(C)**: Western blot of α‐SMA, sm22‐α, calponin and SMMHC in undifferentiated ESCs (ESC) and differentiated ESCs (d3,6,9) with (+) and without (−) 4MU treatment. **(D)**: Luciferase assays were performed at day 3 of ESC‐SMC differentiation, showing suppressed promotor activity for sm22‐α in the presence of 4MU. RLU, relative luciferase unit. **(E)**: Removal of pericellular HA in esSMCs by HYAL treatment was visualized by immunofluorescence staining with a biotinylated HA‐binding probe to localize HA (green) in live cells (top panel) and merged with phase contrast image (bottom panel). **(F)**: RT‐qPCR analysis demonstrates that α‐SMA, calponin, sm22‐α and SMMHC expression in esSMCs is significantly attenuated in the presence of HYAL. **(G)**: Western blot analysis shows that α‐SMA, calponin, sm22‐α and SMMHC expression in esSMCs is significantly attenuated in the presence of 4MU and to a lesser extent HYAL. For densitometry quantification see Supporting Information Figure 6. **(H)**: Immunofluorescence staining of SMC markers, α‐SMA, calponin and sm22‐α in esSMCs that have been differentiated under normal differentiation conditions (control) or treated with 4MU or HYAL. PHs demonstrate that in the presence of 4MU or HYAL, esSMCs retain colony‐like morphology characteristic of undifferentiated ESCs. Bars, 100 μm. Images and blots shown are representative of at least three separate experiments, whereas graphs are shown as mean ± SEM of at least three independent experiments. Analyses based on Student's *t* test. *, *p* < .05; **, *p* < .01, versus control, or two‐way ANOVA, followed by Bonferroni's post test. ^**≠**^, *p* < .001 versus control. Abbreviations: 4MU, 4‐methylumbelliferone; ESC, embryonic stem cell; HA, hyaluronan; HYAL, hyaluronidase; PHs, phase contrast images.

The positive correlation between endogenous HA production and ESC‐SMC differentiation was further investigated by treating cells with a HA synthesis inhibitor, 4MU. As expected, 4MU treatment significantly decreased the total amount of HA secreted into the culture medium compared to untreated ESCs over the differentiation time course (Fig. 2A) and was not shown to be cytotoxic as assessed by cell viability assays (Supporting Information Fig. 4). Critically, RT‐qPCR analysis demonstrated that 4MU treatment significantly attenuated the expression of the SMC markers, α‐sma, calponin and sm22‐α and smmhc in esSMCs at the RNA level (Fig. 2B). Western blot analysis confirmed that the same panel of markers were significantly attenuated in the presence of 4MU from day 6 of differentiation (Fig. 2C). Luciferase gene reporter assays were performed to further determine the role of HA synthesis. 4MU treatment significantly decreased sm22 promoter activity in differentiating ESCs (Fig. 2D), thus supporting the concept that endogenous HA synthesis has a positive effect on sm22 de novo transcription.

The role of the HA pericellular coat in ESC‐SMC differentiation was examined after its digestion with HYAL. Imaging of esSMCs following incubation with a biotinylated HA‐binding probe demonstrated that HYAL treatment successfully removed pericellular HA in mature SMCs (Supporting Information Fig. 5) and esSMCs (Fig. 2E). RTq‐PCR analysis demonstrated that HYAL treatment suppressed induction of SMC marker expression in esSMCs (Fig. 2F). This was also confirmed by Western blot (Fig. 2G). Notably, densitometry quantification revealed that whilst 4MU and HYAL treatment significantly suppresses induction of SMC marker expression, 4MU treatment was superior (Supporting Information Fig. 6). Immunofluorescence staining confirmed that in the presence of 4MU or HYAL, esSMCs lost α‐sma, calponin and sm22‐α expression (Fig. 2H). Phase contrast imaging also revealed that 4MU or HYAL treated esSMCs lost their SMC‐like morphology and retained a rounder spherical‐like colony comparable to undifferentiated ESCs (Fig. 2H). Collectively, these data signify that ESC‐SMC differentiation is dependent on the synthesis of extracellular HA and its assembly into a pericellular coat.

### ESC‐SMC Differentiation is Dependent on HAS2‐Induced HA

**Figure 3:**
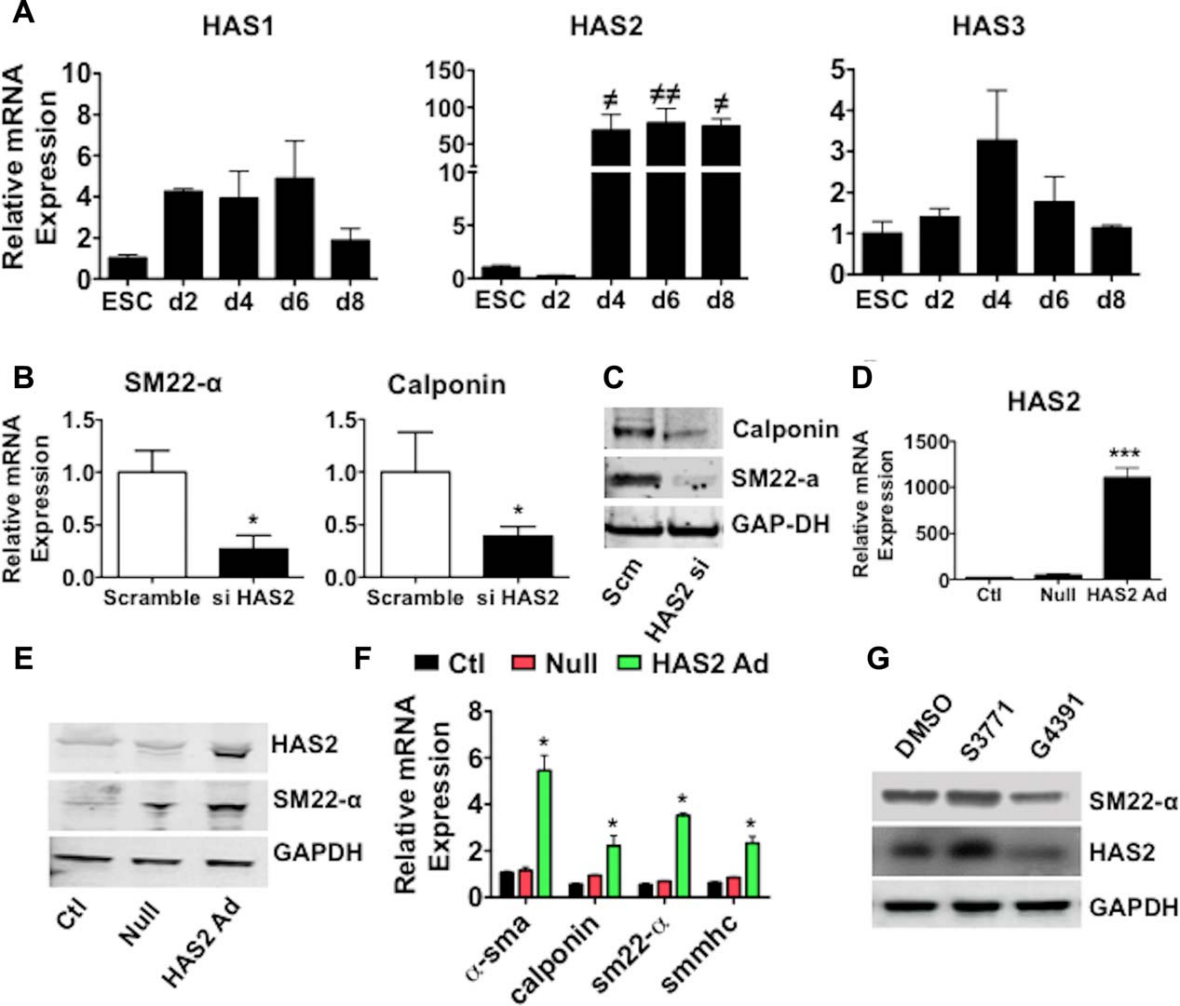
HAS2‐induced hyaluronan is necessary for ESC‐smooth muscle cell (SMC) differentiation. **(A)**: ESCs were seeded on collagen IV‐coated plates (5 μg/ml) and cultured in differentiation medium (DM) for the indicated times. RT‐qPCR analysis shows expression of HAS1/2/3 throughout differentiation time course. Over the course of differentiation HAS2 expression is significantly augmented. ESCs were transfected with HAS2 siRNA or scrambled oligonucleotide control (scramble) for 48 hours prior to further culture in DM for 72 hours. RT‐qPCR **(B)** and Western blot **(C)** show a significant attenuation in expression of SMC markers, sm22‐α and calponin. To further examine the role of HAS2, ESCs were uninfected (ctl) or infected with an adenovirus for HAS2 overexpression (HAS2 Ad) or a null adenovirus (Null) for 48 hours then cultured in DM for a further 72 hours. **(D)**: RT‐qPCR demonstrated successful overexpression of HAS2. This resulted in significant induction of SMC gene expression as assessed by Western blot analysis **(E)** and RT‐qPCR **(F). (G)**: ESCs were pretreated with integrin recognition sequence pentapeptide Glu‐Arg‐Gly‐Asp‐Ser (G4391) for 30 minutes before SMC differentiation on collagen IV‐coated plates (5 μg/ml) and cultured in DM for 4 days. RGD peptide S3771 or DMSO were also used as control treatments. Western blot analysis shows that antagonizing integrin function with G4391 resulted in inhibition of sm22‐α and HAS2 expression. All blots shown are representative of at least three separate experiments, graphs are shown as mean ± SEM of at least three independent experiments. Analyses based on Student's *t* test. *, *p* < .05; ***, *p* < .001, versus scramble/null, or one‐way ANOVA, followed by Dunnett's Multiple Comparison Test. ^**≠**^, *p* < .05; ^**≠≠**^, *p* < .01, versus ESC. Abbreviations: DMSO, dimethyl sulfoxide; ESC, embryonic stem cell.

Having demonstrated the necessity for HA synthesis and pericellular assembly during ESC‐SMC differentiation, we investigated the mRNA expression levels of the three HAS isoforms. The expression of HAS1, 2 and 3 was analyzed by RT‐qPCR in undifferentiated ESCs and at days 2, 4, 6 and 8 of differentiation (Fig. 3A). No significant difference in HAS1/3 expression was observed during differentiation, however, a prominent induction of HAS2 mRNA expression was observed at day 4 (∼70‐fold) and this upregulation was sustained throughout differentiation. A direct comparison between ESCs and esSMCs revealed that whilst HAS 1‐3 levels are similar in undifferentiated ESCs, HAS2 is the dominant synthase isoform induced during SMC differentiation (Supporting Information Fig. 7A). Furthermore esSMC stained positive for HAS2 and not HAS1/3 (Supporting Information Fig. 7B). Based on these findings, HAS2‐induced HA seemed to play a vital role in the regulation of ESC‐SMC differentiation. In parallel experiments, the mRNA expression levels of the three HYAL isoforms (HYAL 1, 2 and 3) which are responsible for the catabolism of HA polymers were analyzed by RT‐qPCR analysis. Expression of HYAL 1 and 2 did not alter over the course of differentiation but HYAL 3 demonstrated significant induction between days 0 and 8 (Supporting Information Fig. 8). To test whether HAS2‐induced HA was necessary for ESC‐SMC differentiation, both overexpression and silencing strategies of HAS2 were used. HAS2 siRNA knockdown experiments in esSMCs demonstrated attenuated expression of the SMC markers, sm22‐α and calponin at the RNA level (Fig. 3B) and protein level (Fig. 3C). The effects of HAS2 overexpression by adenoviral gene transfer were also determined. Successful overexpression of HAS2 (Fig. 2D) demonstrated enhanced expression of sm22‐α at the protein level (Fig. 3E) and induced significant gene expression of the all the SMC markers, α‐sma, calponin, sm22‐α and smmhc at the RNA level (Fig. 3F). Furthermore, impairment of integrin/collagen IV interaction using integrin inhibitor G4391 resulted in inhibition of sm22‐α and HAS2 expression (Fig. 3G). These data provide compelling evidence that collagen‐IV‐mediated ESC‐SMC differentiation is HAS2 dependent.

### HA Dependent ESC‐SMC Differentiation is Mediated Through EGFR and ERK Signaling

**Figure 4:**
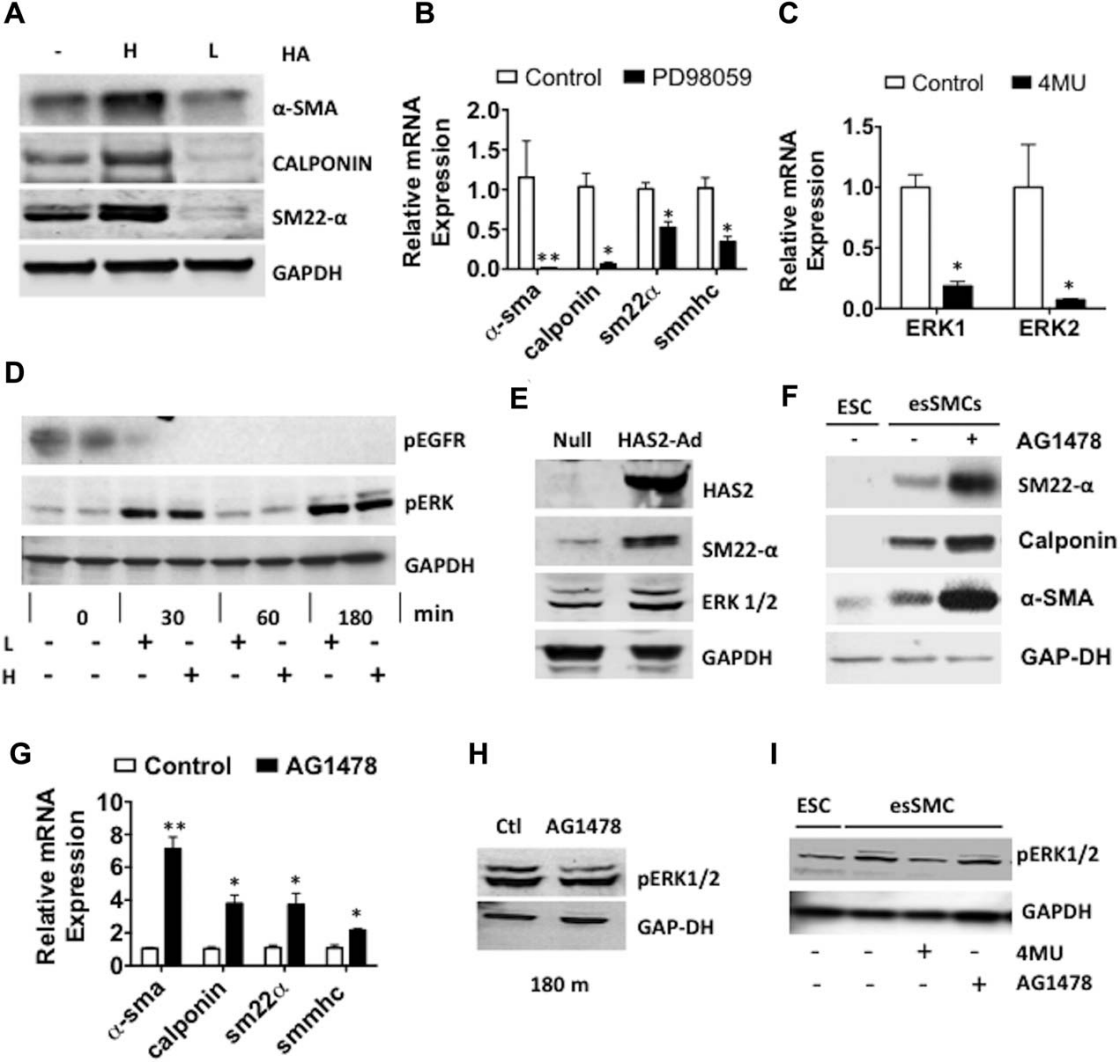
EGFR inactivation and ERK activation mediate ESC‐smooth muscle cell (SMC) differentiation. **(A)**: Western blot of α‐SMA, sm22‐α and calponin in esSMCs that have been differentiated either under normal differentiation conditions (−) or with exogenous addition of HMW‐HA (H) or LMW‐HA (L) (200 μg/ml). HMW‐HA potentiates whereas LMW‐HA attenuates SMC marker expression. **(B)**: RT‐qPCR analysis shows the expression of the SMC markers α‐SMA, calponin, sm22‐α and SMMHC in esSMCs following differentiation with or without the ERK1/2 inhibitor PD98059 (10 μmol/l). **(C)**: RT‐qPCR analysis demonstrates that ERK1 and ERK2 expression in esSMCs is significantly attenuated in the presence of 4MU (0.5 mmol/l). **(D)**: ESCs were stimulated with exogenous LMW‐HA (L) or HMW‐HA (H) at the indicated time points. Cell lysates were analyzed by Western blotting with antibodies against pEGFR and pERK1/2. Undifferentiated ESCs demonstrate basal EGFR activation which is abolished by HA stimulation. Addition of HA displays cyclic ERK1/2 phosphorylation. **(E)**: ESCs were infected with an adenovirus for HAS2 overexpression (HAS2 Ad) or a null adenovirus (Null) for 48 hours then differentiated in differentiation medium (DM) for a further 72 hours. Western blot analysis demonstrated that successful overexpression of HAS2 resulted in induction of sm22‐α and ERK1/2. Western blot analysis **(F)** and RT‐qPCR analysis **(G)** shows the gene expression of the SMC markers α‐SMA, calponin and sm22‐α in esSMCs differentiated in DM alone or in combination with the EGFR inhibitor AG1478 (10 μmol/l). **(H)**: Western blot showing Inhibition of EGFR signaling by AG1478 (10 μmol/l) has no effect on pERK1/2 at 180 m differentiation. **(I)** Western blot showing phosphorylation of ERK1/2 in esSMCs following Inhibition of HA synthesis by 4MU (0.5 mmol/l) and Inhibition of EGFR signaling by AG1478 (10 μmol/l). 4MU suppressed pERK1/2 levels whereas AG1478 had no effect. All blots shown are representative of at least three separate experiments, graphs are shown as mean ± SEM of at least three independent experiments. Analyses based on Student's *t* test. *, *p* < .05; **, *p* < .01, versus control. Abbreviations: 4MU, 4‐methylumbelliferone; ESC, embryonic stem cell; HA, hyaluronan.

Having demonstrated a role for endogenous HA synthesis, this prompted us to explore a role for exogenous HA in ESC‐SMC differentiation. We differentiated ESCs toward SMCs under normal differentiating conditions and in the presence of LMW‐HA or HMW‐HA (Fig. 4A). In esSMCs, the expression of all SMC markers were augmented by the addition of HMW‐HA when compared with standard differentiation medium as evaluated by Western blot analysis. Conversely, the addition of LMW‐HA attenuated the induction of SMC marker expression in esSMCs. The ability of HMW‐HA to promote differentiation was consistent with our HAS data since HAS2 is responsible for synthesizing HMW‐HA only.

The HA‐CD44 signaling pathway is largely associated with EGFR and ERK signaling 18, 19, 41, 42, 43. The role of ERK signaling was investigated by pretreating ESCs with PD98059, a potent and selective cell permeable inhibitor of MEK1/2 which blocks phosphorylation of ERK1/2. RT‐qPCR analysis demonstrated that PD98059 caused a significant reduction in the expression of SMC markers, α‐sma, calponin, sm22‐α and smmhc in esSMCs (Fig. 4B). Inhibition of HA synthesis by 4MU significantly suppressed ERK1 and ERK2 in esSMCs, indicating that ERK signaling was mediated by HA (Fig. 4C). To further investigate the role of HA‐dependent activation of ERK1/2, exogenous LMW‐HA or HMW‐HA was added to the culture medium and phosphorylation of ERK1/2 was assessed over short‐term stimulations. Undifferentiated ESCs (0 min) presented with low levels of pERK1/2. In the presence of LMW‐HA and HMW‐HA, augmented pERK1/2 was detected at 30 minutes and peaked at 180 minutes (Fig. 4D). No notable difference in ERK1/2 activation was observed between treatment with LMW‐HA or HMW‐HA. Interestingly, the activation of ERK1/2 by HA appeared to be cyclic and returned to basal levels at 60 minutes before peak phosphorylation at 180 minutes. We investigated the regulatory role of HAS2‐induced HA synthesis in ERK1/2 activation by HAS2 adenoviral gene transfer in esSMCs. Western blot analysis demonstrated that prominent HAS2 induction enhanced sm22‐α expression in parallel with elevated ERK1/2 activation (Fig. 4E). Interestingly, the detection of basal EGFR phosphorylation in undifferentiated ESCs was abolished following stimulations with LMW‐HA and HMW‐HA throughout the time course (Fig. 4D). To investigate the role of EGFR signaling further, ESCs were differentiated toward SMCs in the absence or presence of AG1478, a tyrosine kinase inhibitor that works as a potent and selective inhibitor of EGFR phosphorylation. Western blot (Fig. 4F) and RT‐qPCR (Fig. 4G) analysis revealed that esSMCs treated with AG1478 demonstrate a significant increase in expression of SMC markers, α‐sma, calponin, sm22‐α and smmhc compared to untreated esSMCs. Treatment of esSMCs with AG1478 however had no effect on early (Fig. 4H) or late (Fig. 4I) ERK1/2 phosphorylation suggesting that activation of ERK is not dependent on EGFR signaling. Western blot analysis confirmed that upregulated ERK phosphorylation in esSMCs is restored to basal levels displayed in undifferentiated ESCs when treated with 4MU, demonstrating the importance of HA‐dependent activation of ERK1/2 in ESC‐SMC differentiation (Fig. 4I). Collectively, these data suggest that HA dependent activation of ERK1/2 mediates ESC‐SMC differentiation. Also, sustained basal EGFR signaling may act as a barrier for initiating differentiation, which is overcome by HA‐dependent signaling. However, both HA‐mediated events appear to be independent from one another.

### HAS2 Induced HA‐CD44 Interaction with EGF‐R Drives ESC‐SMC Differentiation

**Figure 5:**
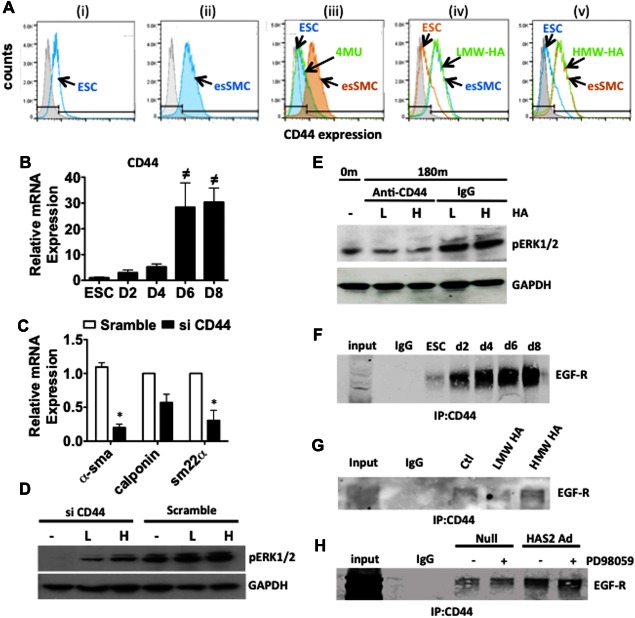
HA mediated CD44‐EGFR interaction drives ESC‐smooth muscle cell (SMC) differentiation. **(A)**: FACS analysis showing expression of HA cell surface receptor CD44. The percentages of positive cells were calculated based on the isotype controls (gray plot). Only 24.8% of ESCs expressed CD44 (i) whereas following differentiation, esSMC expressed 85.4% (ii). Treatment with 4MU (0.5 mmol/l) during differentiation restores CD44 levels back to that of undifferentiated ESCs (iii). During differentiation of ESCs the addition of either exogenous LMW‐HA (iv) or HMW‐HA (v) treatment (200 μg/ml) did not alter CD44 expression. FACS data representative for at least three independent experiments with similar results are shown. **(B)**: ESCs were seeded on collagen IV‐coated plates (5 μg/ml) and cultured in DM for the indicated times. RT‐qPCR analysis shows augmented gene expression of CD44 over the course of ESC‐SMC differentiation. **(C)**: To further examine the role of CD44, ESCs were transfected with CD44 siRNA or scrambled oligonucleotide control (scramble) for 48 hours prior to further culture in DM for 72 hours. RT‐qPCR shows a significant attenuation in SMC markers, α‐SMA, calponin and sm22‐α. **(D)**: ESCs were transfected with CD44 siRNA or scrambled oligonucleotide control (scramble) for 48 hours prior to further differentiation for 72 hours in DM alone (−) or stimulation with exogenous LMW‐HA (L) or HMW‐HA (H) (200 μg/ml). Western blot analysis demonstrated that knockdown of CD44 suppressed basal and HA‐mediated ERK1/2 activation. **(E)**: ESCs were preincubated with an anti‐CD44 antibody (5 μg/ml) or IgG control then differentiated in the presence of either LMW‐HA (L) or HMW‐HA (H) (200 μg/ml) for 120 m. Western blot analysis demonstrates that HA mediated pERK1/2 is retarded in the presence of anti‐CD44 antibody. **(F‐H)**: For all experiments samples were IP with anti‐CD44 antibody, followed by immunoblotting with anti‐EGFR antibody. Western blot analysis of the precipitated complexes revealed marked binding of CD44 with EGFR during the course of differentiation (d2‐d8) compared to undifferentiated ESCs (ESC) (F). CD44‐EGFR co‐localisation of esSMCs (ctl) was enhanced in the presence of HMW‐HA but not LMW‐HA (G). ESCs were infected with an adenovirus for HAS2 overexpression (HAS2 Ad) or a null adenovirus (Null) for 48 hours then differentiated toward SMCs in the absence or presence of the ERK1/2 inhibitor PD98059 (10 μmol/l). HAS2 overexpression resulted in enhanced CD44 and EGFR association which was unaltered by ERK1/2 inhibition (H). All blots shown are representative of at least three separate experiments, graphs are shown as mean ± SEM of at least three independent experiments. Analyses based on Student's *t* test. *, *p* < .05, versus scramble, or one‐way ANOVA, followed by Dunnett's Multiple Comparison Test. ^**≠**^, *p* < .01, versus ESC. Abbreviations: 4MU, 4‐methylumbelliferone; DM, differentiation medium; ESC, embryonic stem cell; FACS, fluorescence‐activated cell sorting; HA, hyaluronan; HMW‐HA, high molecular weight‐hyaluronan; LMW‐HA, low molecular weight‐hyaluronan; IP, immunoprecipitated.

FACS analysis was used to investigate the role of cell surface CD44 in ESC‐SMC differentiation. We found that 85.4% of esSMCs expressed CD44 in comparison to only 24.8% in undifferentiated ESCs. Furthermore, in the presence of 4MU, levels of CD44 expression in esSMCs returned to those exhibited in undifferentiated ESCs, however, application of exogenous LMW‐HA or HMW‐HA during differentiation of ESCs caused no additional CD44 induction (Fig. 5A). Analysis of CD44 gene expression by RT‐qPCR confirmed significant upregulation during ESC‐SMC differentiation (Fig. 5B). Silencing CD44 in siRNA transfection experiments illustrated the essential role that CD44 plays in ESC‐SMC differentiation. Successful suppression of CD44 gene expression significantly attenuated the induction of SMC markers, α‐sma, calponin and sm22‐α in esSMCs (Fig. 5C). Following transfection of CD44 siRNA in esSMCs, phosphorylation of ERK1/2 was suppressed compared to scramble control. Furthermore, the increase in pERK1/2 by LMW‐HA or HMW‐HA stimulation was suppressed following CD44 silencing (Fig. 5D). To confirm the role of CD44 in early activation of ERK1/2, ESCs were differentiated in the presence of an anti‐CD44 antibody or dimethyl nsulfoxide as a control with or without HA treatment. After 120 minutes, induction of pERK1/2 by LMW‐HA and HMW‐HA was notably reduced in the presence of anti‐CD44 antibody (Fig. 5E). Anti‐CD44 immunoprecipitation followed by anti‐EGFR immunoblot analysis revealed that there is an increase in the binding between EGFR and CD44 during SMC differentiation from ESCs (Fig. 5F). This binding was shown to be regulated by HA since the treatment of esSMCs with exogenous HMW‐HA enhanced CD44‐EGFR association. However, addition of exogenous LMW‐HA failed to promote further binding (Fig. 5G). Using a HAS2 adenovirus, we demonstrated that overexpression of HAS2 resulted in enhanced EGFR accumulation in the CD44 immunoprecipitant compared to null transfected ECSs (Fig. 5H). CD44‐EGFR association was unaltered by the ERK1/2 inhibitor PD98059, thus implying that HAS2‐induced HA activation of ERK played no role in facilitating this complex which also corroborates with earlier data (Fig. 4H‐I).

### HA Niche Promotes Neovascularization and Neointima Formation

**Figure 6:**
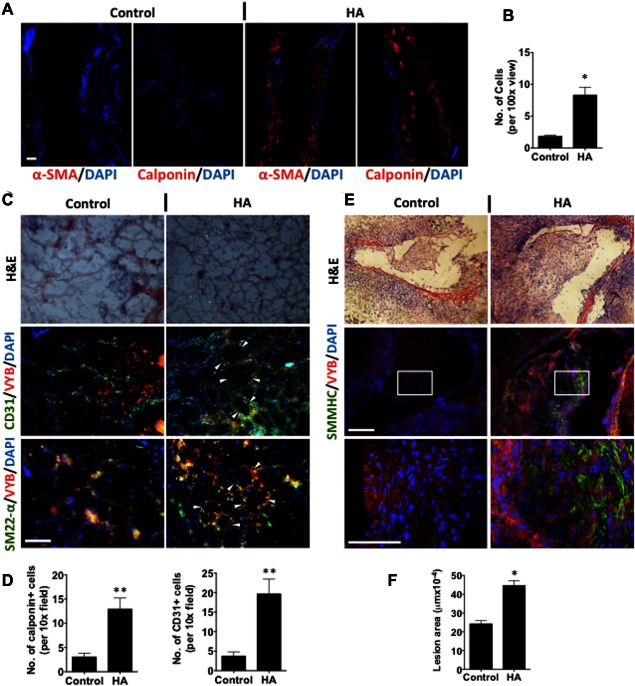
HA niche promotes esSMCs neointimal accumulation and neovascularization. **(A)**: Embryonic stem cells (5 × 10^5^) were seeded in the outer layer of a decellularized mouse thoracic aorta in a bioreactor system, either in the presence (HA) or absence (Control) of HMW‐HA in the circulating medium followed by application of shear stress. After 1 week, the vessels were harvested and embedded in mouse liver for nitrogen freezing. The frozen sections were subjected to immunofluorescence staining for the smooth muscle cell (SMC) markers α‐SMA, and calponin. Graph **(B)** represents mean ± SEM from three independent experiment. **(C)** esSMCs (5 × 10^5^) maintained in normal differentiation medium (control) or HMW‐HA (HA) were mixed with esECs (5 × 10^5^) then added to a Matrigel plug before subcutaneous injection into a LacZ mouse. After 1 week the Matrigel plug was removed and sections were stained for EC marker CD31 or SMC marker sm22‐α (green). esSMCs were labeled with Vybrant (red) before implantation in order to be distinguished from esECs. In control samples esECs stained positive for CD31 but exhibited weak interaction with the vybrant stained esSMCs. In HA treated samples vybrant stained esSMCs displayed enhanced sm22‐α co‐localization (yellow) and interacted with esECs to form substantially denser and more robust tube‐like structures (indicated by arrows). **(D)**: The number of tubes that were either calponin or CD31 positive were quantified in five random fields of view at x10 magnification, mean ± SEM *n* = 6 animals. **(E)**: Vena cava segments were surgically removed from C57BL/6 mice under anesthesia and subsequently grafted into carotid arteries of apoE‐deficient recipient mice. esSMCs (5 × 10^5^) were stained with a Vybrant cell tracker dye before being seeded onto the adventitial side to envelop the vein grafts. Grafted tissue fragments were harvested 2 weeks after surgery and stained with H&E and immunofluorescence stained with SMMHC and DAPI. Vein segments were seeded with esSMCs that were differentiated in differentiation medium in the absence of (control) or presence of HMW‐HA (HA). White squares represent the neointima, and the areas of neointimal leisions in vein grafts were quantified as described in methods section. Graph **(F)** represents mean ± SEM, six animals per group. Bars, 100 μm. All analyses based on Student's *t* test. *, *p* < .05; **, *p* < .01, versus control. Abbreviations: HA, hyaluronan; H&E hematoxylin and eosin.

To test whether HMW‐HA (HA) can also promote SMC differentiation that may have functions ex vivo and in vivo, three experimental models were used. First, a bioreactor was established by using a mouse decellularized vessel, in which ESCs were applied to the external side of the vessel and HA added into the circulating medium (Supporting Information Fig. 9). DM in the absence of HA was used as a control. Although a small number of cells in the vessel wall, in the absence of exogenous HA, displayed SMC positivity, the addition of HA significantly enhanced the number of α‐SMA + / calponin + cells in the decellularized vessels over multiple layers (Fig. 6A, 6B). The second model used to test the function of HA‐stimulated SMCs was in vivo vasculogenesis using a matrigel plug assay. We have previously reported that esECs can form vascular tubes in vitro and in vivo 44. esSMCs maintained in normal differentiation medium (control) or HMW‐HA (HA) were labeled with Vybrant cell tracker and mixed with esECs before subcutaneous injection into a LacZ mouse. After 7 days the plug was removed and sections were stained. Although esECs stained positive for EC marker CD31, they exhibited a poor interaction with the Vybrant stained esSMCs in the control experiment. However, in HA treated samples, esSMCs displayed increased staining for the SMC marker, sm22‐α and interacted with esECs to form substantially denser and more robust three dimensional networks of vascular‐like structures, in which some large vessels were found (Fig. 6C, 6D). Lack of X‐gal staining confirmed that the infiltration of cells into the plug were donor‐ and not host‐derived (Supporting Information Fig. 10). In the third model, we used a mouse vein graft to compare the potential of neointimal formation of esSMCs differentiated with or without (control) exogenous HA. Both cell populations were applied to the external side of vein grafts after isografting into apolipoprotein E‐deficient mice. Grafted tissue fragments were harvested 2 weeks post surgery and stained with immunofluorescence smmhc to indicate SMC accumulation. Implanted cells were also stained with Vybrant tracking dye to discriminate between the contribution of donor and recipient cells. Grafts that had been seeded with esSMCs maintained in a HA microenvironment presented distinctly larger lesions with enhanced double staining of smmhc and Vybrant cell tracker dye compared to the control (Fig. 6E, 6F). They also exhibited increased HA deposition (Supporting Information Fig. 11). These results suggested that HA stimulation resulted in a higher proportion of esSMCs to accumulate into the graft with an enhanced SMC phenotype, and enhanced endogenous HA secretion.

## Discussion

Regulation of stem cell homeostasis is mediated in part through extrinsic cues resulting from interactions between cells and the ECM within the niche 5, 7. Despite being identified as a niche component 12, 33, 45, 46, the autocrine role of endogenous HA during stem cell differentiation is a complicated and inadequately defined process. We report that dynamic changes in HA homeostasis are associated with ESC‐SMC differentiation. Undifferentiated ESCs secreted relatively low amounts of extracellular HA with no evident pericellular retention. Differentiation (at as early as day three) coincided with a dramatic increase in HAS expression, accumulation of extracellular HA and its assembly into pericellular coats. Detailed analysis of the temporal pattern of HA synthesis revealed that while an accumulative increase in HA is observed globally, following an early and sizeable induction of secreted HA (day 3), synthesis is rapidly muted. These data suggest that an early induction of HA synthesis is sufficient to propagate differentiation without the need for persistent secretion. This could be simply explained through an autocrine feedback mechanism. Assuming endogenous HA production and pericellular attachment via CD44 ensues early on, the need for additional HA synthesis would be obsolete, providing pericellular HA and the available pool of extracellular HA is not degraded/depleted. Nevertheless, 4MU treatment ablated SMC differentiation at later time points and suppressed sm22‐α promoter activity in esSMCs, confirming that the presence of extracellular HA is required throughout differentiation. In fact, only conditioned medium from day 3 differentiated cells provided effective SMC induction when used to culture ESCs, which was most likely a simple reflection of increased HA concentration.

4MU is commonly described as a specific inhibitor of HA synthesis, however, it was recently reported that 4MU may impact on other GAGs, albeit to a lesser extent 47 so we cannot rule out this phenomenon in our study. Crucially, we demonstrate that in addition to using 4MU, specific stripping of the HA coat using HYAL treatment attenuates SMC differentiation. Mature SMCs demonstrated extensive pericellular HA coats as previously reported 25 and likewise esSMCs exhibited HA coats, albeit smaller. The magnitude of pericellular HA accumulation has been shown to closely parallel the differentiation status of cells. For example, in mesenchymal stem cells, CD44‐dependent HA coats may provide a protective niche that support “stemness” 8. In the current study, we demonstrate that following HA synthesis, retention of pericellular HA is a critical step for the generation of esSMCs.

Intracellular HA is now well documented in many cell types including SMCs 48. Recent data present a role for intracellular HA in mediating cytoskeletal elements 41, 49, 50. It has been proposed that the source of intracellular HA is either degraded pericellular material 51 or from intracellular stores 52. As far as we know, this is the first time intracellular HA has been documented in undifferentiated ESCs but given the fact that they demonstrated no pericellular content the latter explanation seems more plausible. In any case, intracellular HA dissipated during differentiation but we did not investigate its significance. Despite observing a reduction in HYAL2 expression, the significant increase in HYAL3 levels may offer a mechanism for HA degradation, however, the regulation of intracellular HA during ESC‐SMC differentiation and the role of HYALs is unclear and warrants further investigation.

As previously reported 34, we demonstrated that all three HAS isoforms were expressed in undifferentiated and differentiating ESCs. Notably, HAS2 was the primary synthase isoform induced. We were able to demonstrate that knockdown and forced overexpression of HAS2 resulted in suppression and elevation of SMC markers, respectively, highlighting the intrinsic role for HAS2 in ESC‐SMC differentiation. Our findings are consistent with a previous study that reports that hESC differentiation is facilitated by HAS2‐induced HA synthesis 9. However, the exact mechanism and signaling pathways involved have remained unclear. Here we propose that through binding to its principle receptor, CD44, HAS2‐induced HA modulates EGFR and ERK1/2 signaling.

A recent study demonstrated that the extent of CD44 expression regulates differentiation in CD34^+^ progenitors 53. We demonstrated that there was a prominent increase in the expression of cell surface CD44 in esSMCs compared to undifferentiated ESCs. Interestingly, endogenous HA synthesis was found to directly augment CD44 levels since 4MU treatment repressed CD44 induction in esSMCs. This may be explained by the theory that inhibition of HA synthesis prevents retention at the cell surface with its synthase, or interaction with HA receptors, CD44 and RHAMM 54. These receptors can readily respond to changes in amounts and size of cell surface HA, and transmit signals intracellularly 55 which may regulate expression of the receptor (i.e., CD44) itself. Nonetheless, the addition of exogenous HA did not demonstrate further induction of CD44 expression in esSMCs possibly due to saturated levels. Having demonstrated that HAS2‐induced HA synthesis is required for ESC‐SMC differentiation, critically, using an siRNA strategy we demonstrated that silencing CD44 attenuated ESC‐SMC differentiation, suggesting that HA mediates its effect through its principle receptor CD44.

The EGFR has been shown to be a regulator of “stemness” in cancer cells. It was recently reported 56 that under normal development constitutive EGFR becomes inactivated as cancer stem cells differentiate and the current study supports this model. We demonstrate that undifferentiated ESCs exhibit basal levels of pEGFR and the addition of exogenous HA was sufficient to abolish basal activity at as early as 30 minutes. Furthermore, inhibition of EGFR tyrosine kinase by AG1478 resulted in upregulation of SMC marker expression. Collectively these results suggest that the EGFR in its active form plays a critical role in maintaining pluripotency, supporting its role as a regulator of “stemness” in ESCs. Mechanistically, loss of EGFR activation coincided with increased binding to CD44, which was further augmented by addition of HMW‐HA either exogenously or via HAS2 overexpression. These results imply that HAS2‐induced HA mediates the functional coupling of CD44 with the EGFR resulting in EGFR inactivation.

**Figure 7:**
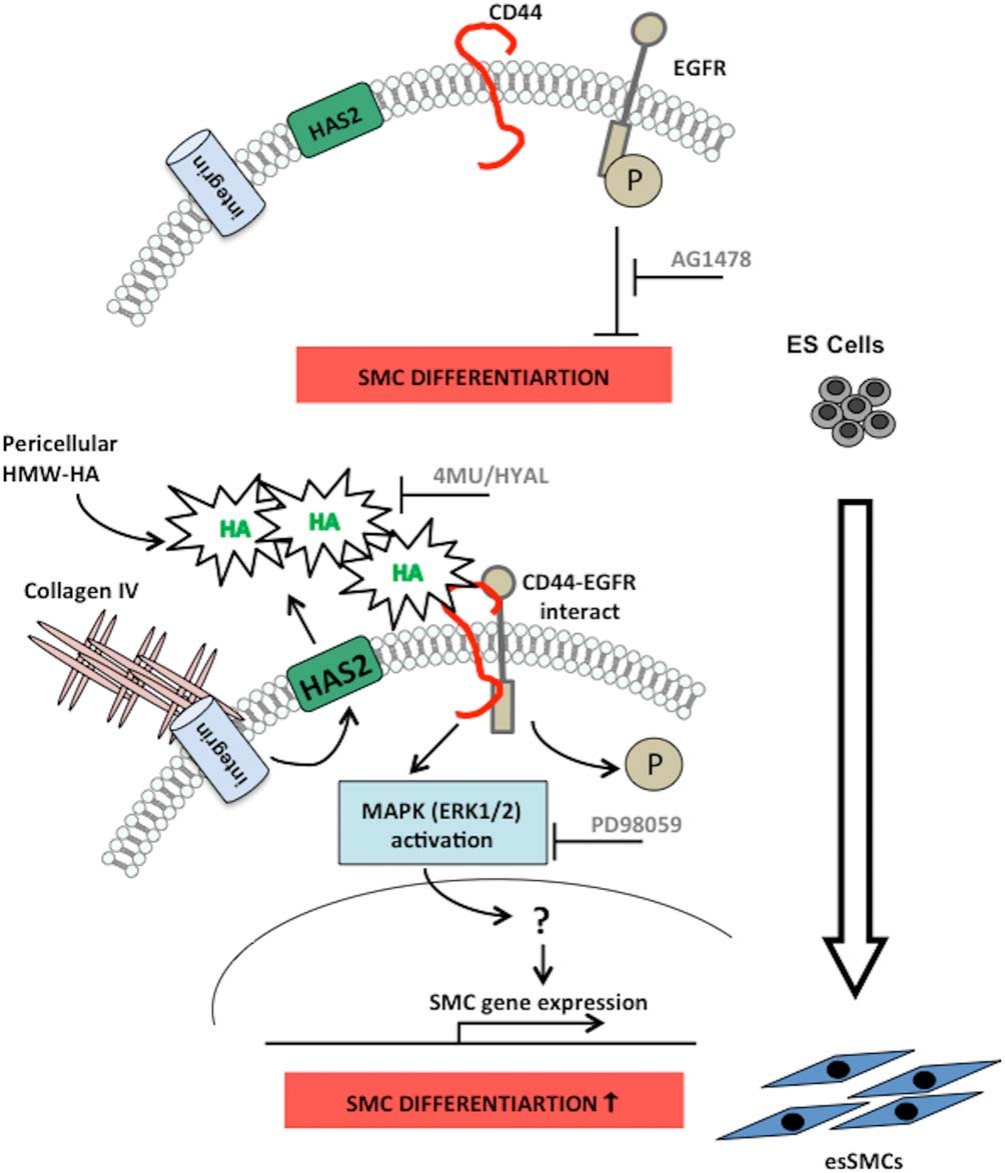
Illustrates our current proposed model for HA‐mediated embryonic stem cell (ESC)‐smooth muscle cell (SMC) differentiation. Undifferentiated ESCs display basal EGFR phosphorylation, which plays a role in maintaining ESC pluripotency. ESCs integrin/collagen IV interaction was associated with HAS2‐dependent extracellular HA generation and its assembly into a pericellular coat. Inhibition of HA synthesis by 4MU or removal of the HA coat by HYAL led to abrogation of SMC gene expression. Binding of HA to its receptor CD44 facilitates its association with and inactivation of the EGFR and activation of ERK1/2, which may translocate to the nucleus and activate transcription factors. Both of these pathways are independent but necessary for stem cell differentiation toward a SMC lineage. Abbreviations: 4MU, 4‐methylumbelliferone; ES, embryonic stem; HA, hyaluronan; HMW‐HA, high molecular weight‐hyaluronan; HYAL, hyaluronidase.

More recently, studies have observed that the HA‐CD44 signaling pathway is largely associated with activation of ERK 18, 42. In addition to loss of EGFR phosphorylation, HA stimulation resulted in rapid phosphorylation of ERK1/2 of a cyclic nature. Inhibition of SMC markers using the ERK inhibitor, PD98059, clearly demonstrated the significance of this pathway in ESC‐SMC differentiation. Consistent with our data, previous work has demonstrated that ERK1/2 activation negatively regulates ESC self‐renewal 57 and drives ESC‐SMC differentiation 4. esSMCs demonstrated augmented levels of activated ERK1/2 compared to undifferentiated ESCs. This was shown to be dependent on endogenous HA synthesis as 4MU treatment abolished elevated levels in esSMCs. Furthermore, HAS2‐induced HA appears to play a role since forced overexpression of HAS2 in esSMCs displayed an increase in ERK1/2 activation. We demonstrated that phosphorylation of ERK1/2 by HMW‐HA (endogenous and exogenous) was mediated through CD44. Silencing of CD44 attenuated early and late ERK activation. This HA‐dependent CD44‐ERK signaling pathway is however distinct from HA‐dependent CD44‐EGFR association, as inhibition of EGFR activity using AG1478 had no effect on early and late ERK activation. When phosphorylated, ERK may translocate to the nucleus and activate transcription factors such as signal transducer and activator of transcription 3, which have been reported to facilitate expression of HAS2, HA synthesis and CD44 expression 58. Figure 7 illustrates our proposed model for HA‐mediated ESC‐SMC differentiation.

The potential for using stem cells in cell‐based vascular therapy holds the promise of permanent, effective treatment for many vascular diseases. Whether stimulating the expansion of endogenous cells or transplanting cells into patients, it is clear that engineering appropriate microenvironments that direct vascular differentiation is paramount. Exploiting the properties of HA has recently led to the design and development of HA‐based hydrogel scaffolds for use in regenerative medicine applications 38. In the present study, we demonstrated that HA can promote formation of SMC layers in a decellularized vessel scaffold, which could be useful for creation of tissue‐engineered vessels. Furthermore, esSMCs maintained in a HMW‐HA rich microenvironment were successfully combined with esECs to form enhanced vascular‐like structures, highlighting how manipulation of the HA niche may present an alternative and attractive strategy for promoting angiogenesis/vasculogenesis. In the formation of neointimal lesions of the vessel wall, lineage‐tracing studies have revealed that the differentiation of vascular stem cells also contributes to vascular remodeling and diseases 59. Thus, the fate of stem cell differentiation into SMCs is a key issue for the progression of vascular diseases. We have previously demonstrated that adventitia stem cells actively contribute to SMC accumulation within the neointima of vein grafts 60. In the current study, esSMCs applied to the external side of vein grafts participated in lesion formation. Significantly, we observed that esSMCs cultured in a rich HMW‐HA microenvironment acquire an augmented SMC phenotype and form distinctly larger lesions, highlighting that HA‐differentiated stem cells can promote neointimal formation.

## Conclusion

We present compelling evidence illustrating that HAS2‐induced HA synthesis and pericellular retention is essential for ESC‐SMC differentiation. Our proposed mechanism provides new insights not only into the mode by which endogenous HA acts as a signal integrator to facilitate ESC‐SMC differentiation but also by remodeling their HA niche, how stem cells can acquire a more susceptible phenotype that promotes vasculogenesis and lesion formation in vein grafts. The possibility of controlling stem cell fate by manipulating the concentration, molecular weight or organization or HA within the niche directly, or by targeting the HA mediated pathways described here may provide significant improvement for clinical therapy in vascular diseases.

## Author Contributions

R.M.L.S.: conception and design, collection and/or assembly of data, data analysis and interpretation, manuscript writing; X.H., M.M.W., E.K., S.I.B., Y.H.: collection and/or assembly of data; D.W.: collection and/or assembly, final approval of manuscript; W.K.: data analysis and interpretation; Q.X.: conception and design, data analysis and interpretation, manuscript writing, final approval of manuscript.

## Disclosure of Potential Conflicts of Interest

The authors indicate no potential conflicts of interest.

## Supporting information

Supplementary Information 1

Supplementary Information 2

Supplementary Information Figure 1

Supplementary Information Figure 2

Supplementary Information Figure 3

Supplementary Information Figure 4

Supplementary Information Figure 5

Supplementary Information Figure 6

Supplementary Information Figure 7

Supplementary Information Figure 8

Supplementary Information Figure 9

Supplementary Information Figure 10

Supplementary Information Figure 11

Supplementary Information Table 1

Supplementary Information Video 1
