# Expression of Concern: Hyaluronan Is Crucial for Stem Cell Differentiation into Smooth Muscle Lineage

**DOI:** 10.1093/stmcls/sxag050

**Published:** 2026-09-22

This is an Expression of Concern regarding: Russell M.L. Simpson, Xuechong Hong, Mei Mei Wong, Eirini Karamariti, Shirin Issa Bhaloo, Derek Warren, Wei Kong, Yanhua Hu, Qingbo Xu, Hyaluronan Is Crucial for Stem Cell Differentiation into Smooth Muscle Lineage, *Stem Cells*, Volume 34, Issue 5, May 2016, Pages 1225–1238, https://doi.org/10.1002/stem.2328

In July 2026, a reader contacted the journal about apparent similarities between Figures 3G and 4F, which were also raised via a PubPeer post (https://pubpeer.com/publications/8356EB6F6190BEA96FB1CD8C674335). The journal is investigating these concerns in line with COPE guidance and is publishing this Expression of Concern to alert readers while the outcome of the corresponding author’s institution investigation is pending.

