# Supplementary figures and images for: Hyaluronan Is Crucial for Stem Cell Differentiation into Smooth Muscle Lineage
Source: Stem Cells. 2016 Mar 4;34(5):1225–38. doi: 10.1002/stem.2328 (PMC4864761; doi:10.1002/stem.2328)

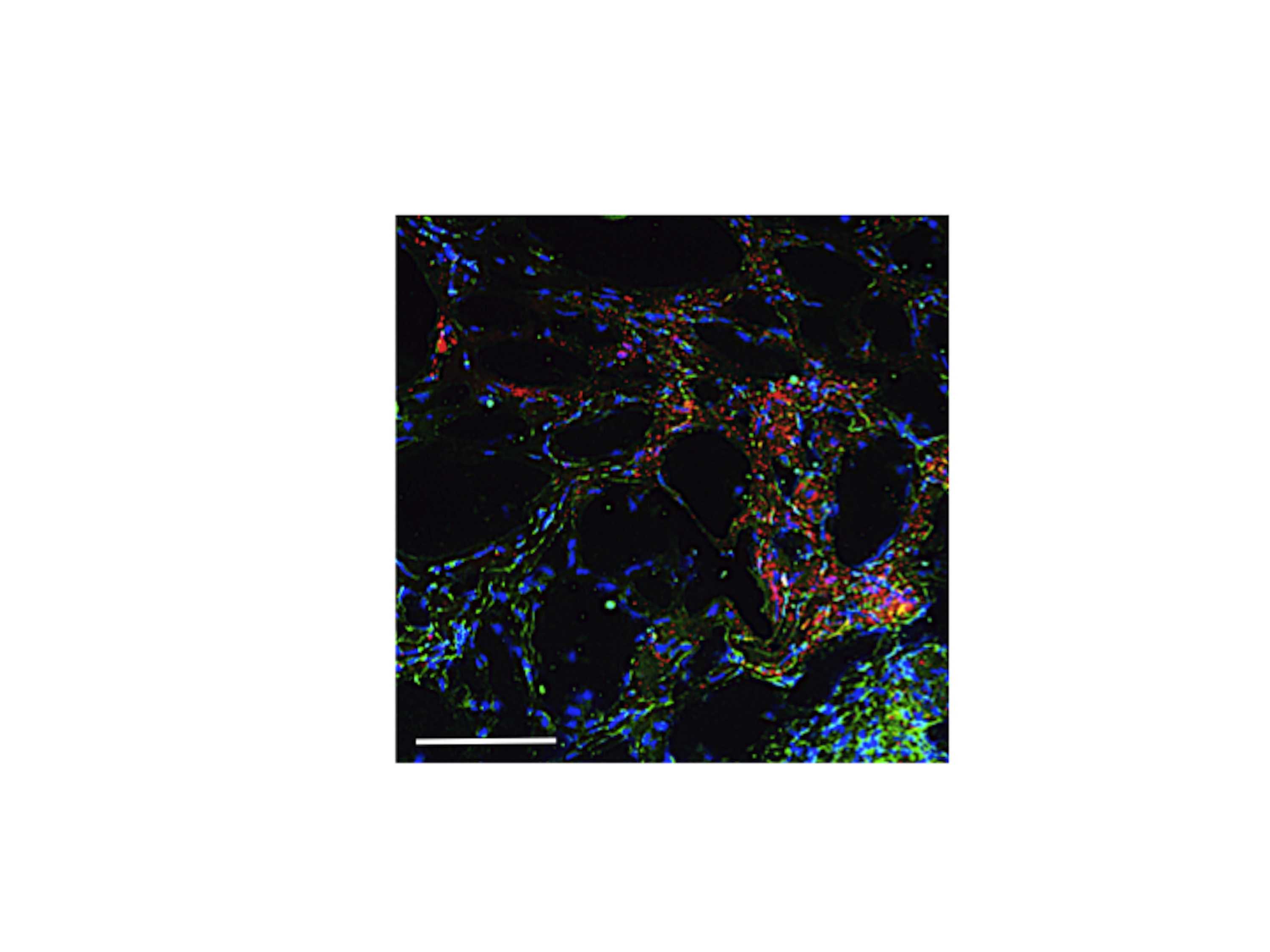

Supplement: Supplementary file 1 — Supplementary Information 1 [file STEM-34-1225-s001.tiff]

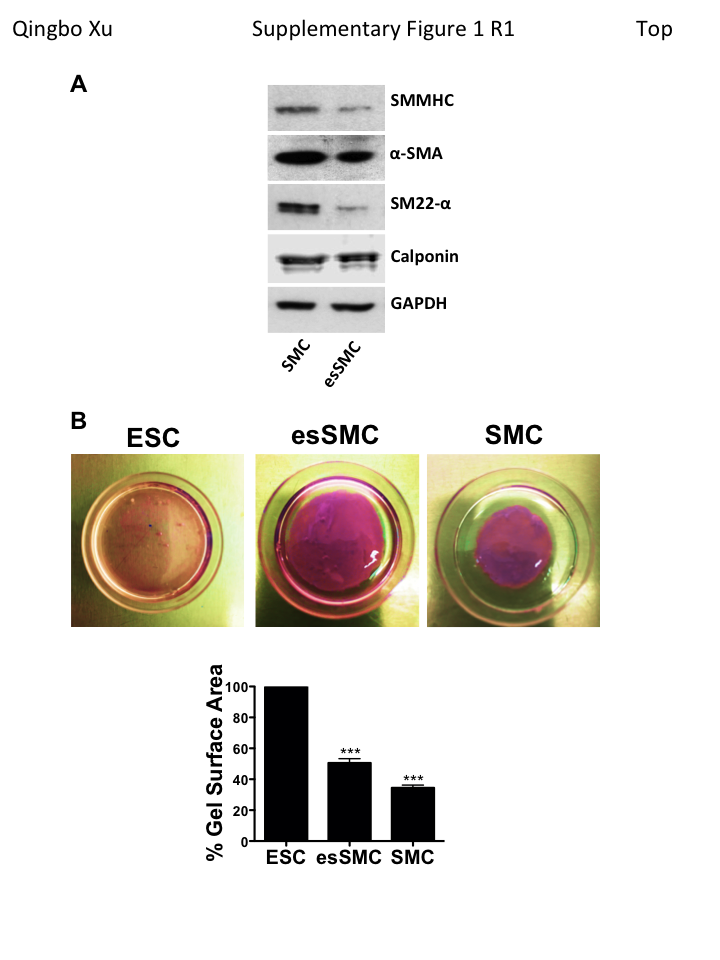

Supplement: Supplementary file 3 — Supplementary Information Figure 1 [file STEM-34-1225-s003.tiff]

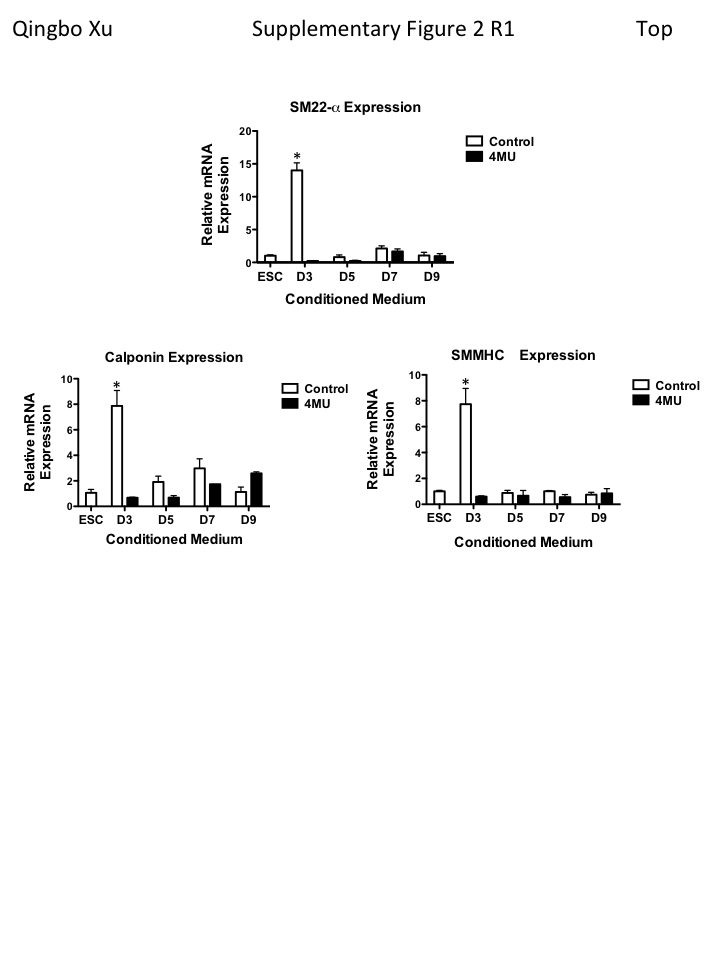

Supplement: Supplementary file 4 — Supplementary Information Figure 2 [file STEM-34-1225-s004.tiff]

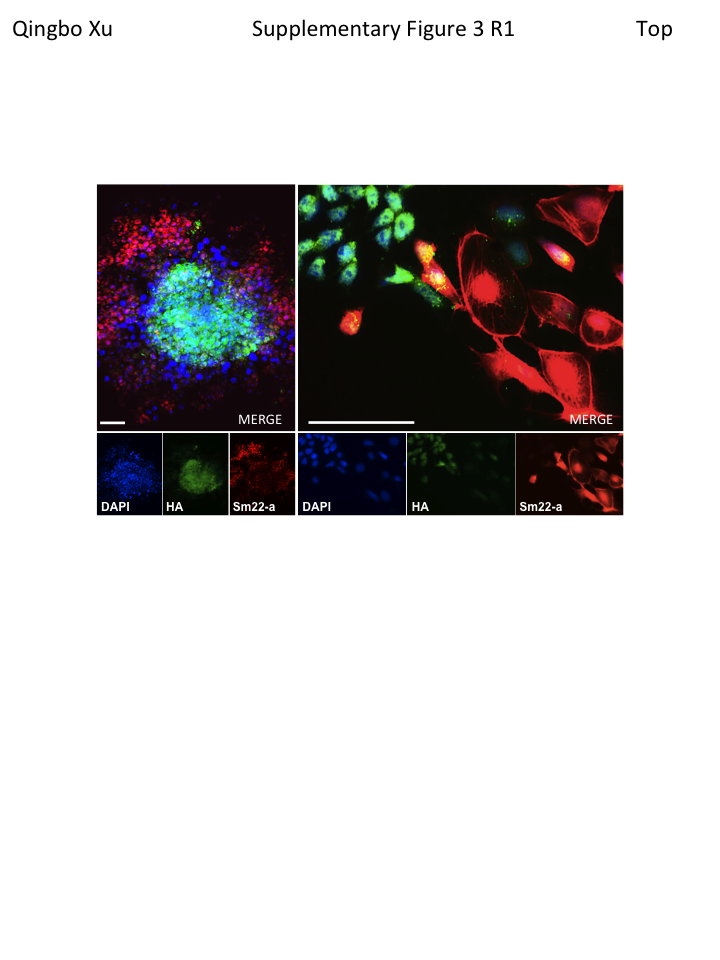

Supplement: Supplementary file 5 — Supplementary Information Figure 3 [file STEM-34-1225-s005.tiff]

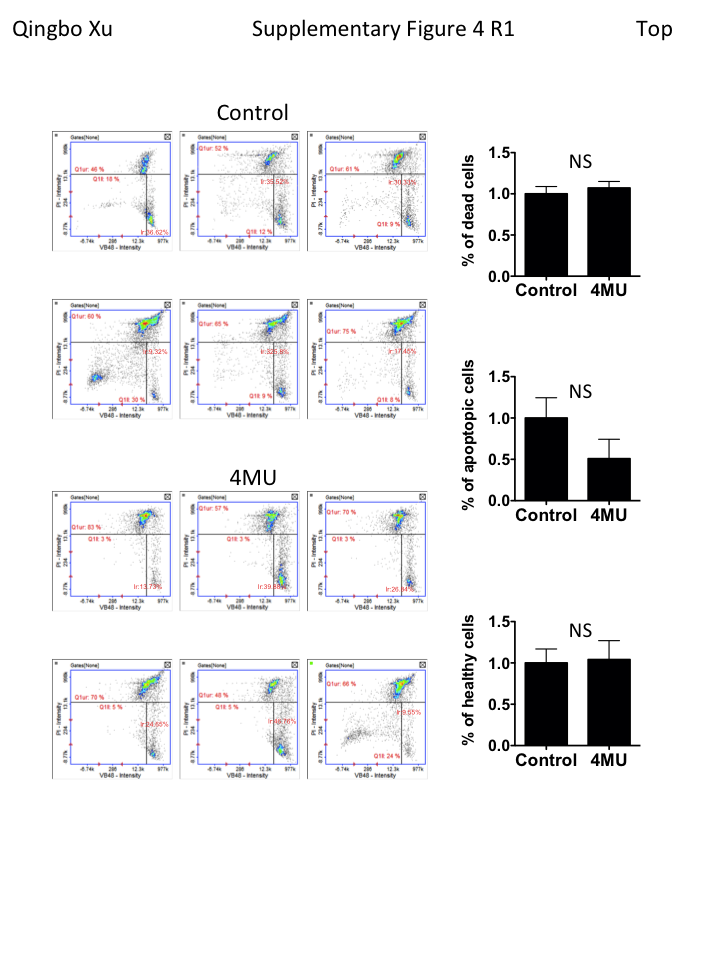

Supplement: Supplementary file 6 — Supplementary Information Figure 4 [file STEM-34-1225-s006.tiff]

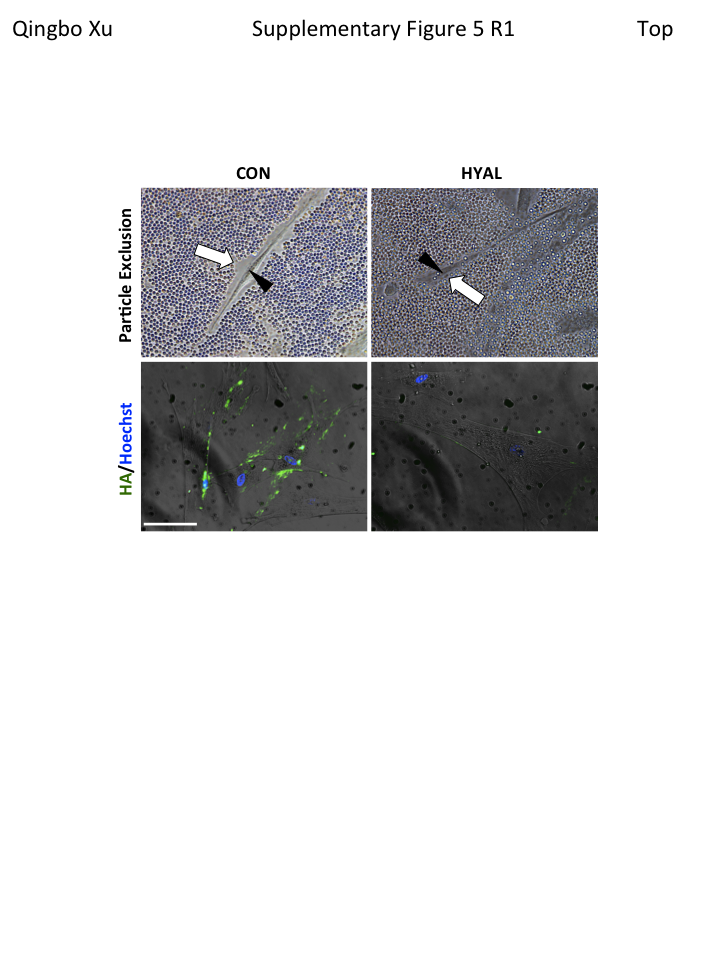

Supplement: Supplementary file 7 — Supplementary Information Figure 5 [file STEM-34-1225-s007.tiff]

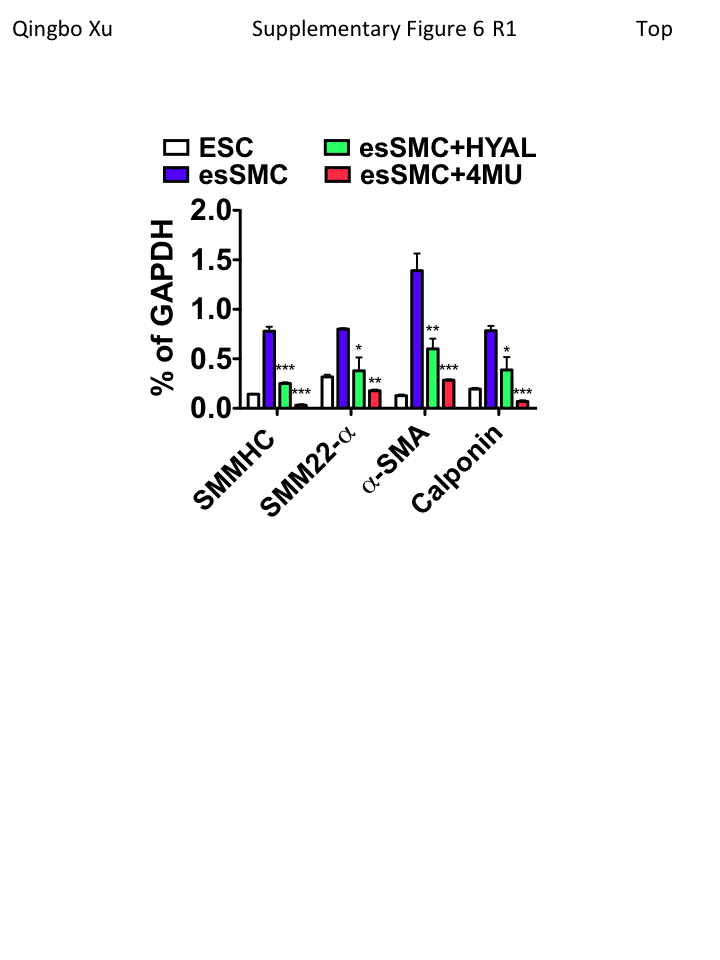

Supplement: Supplementary file 8 — Supplementary Information Figure 6 [file STEM-34-1225-s008.tiff]

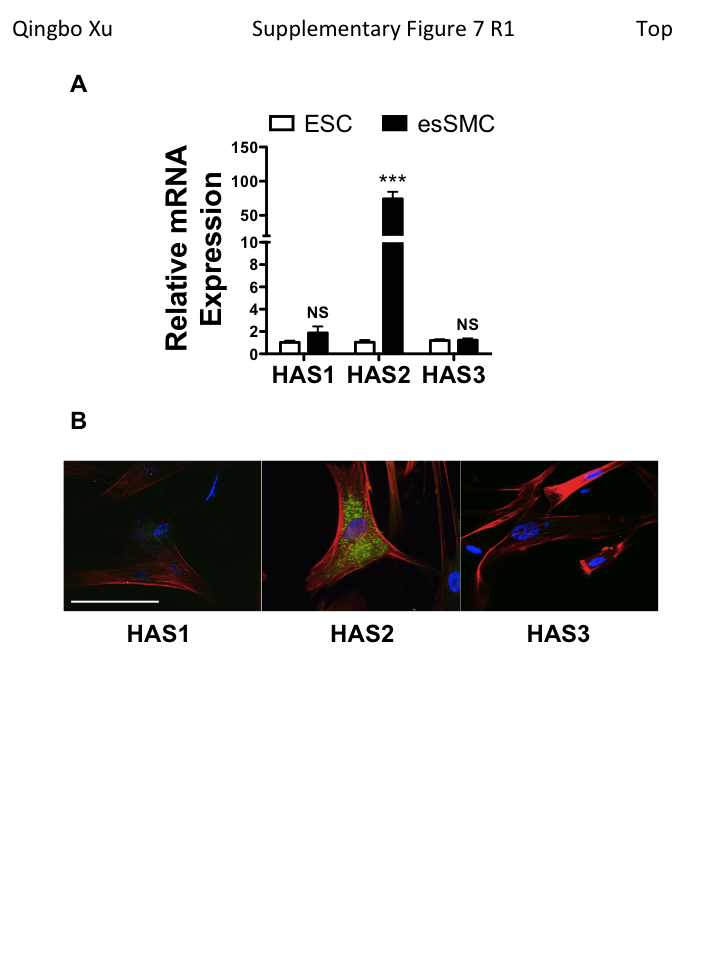

Supplement: Supplementary file 9 — Supplementary Information Figure 7 [file STEM-34-1225-s009.tiff]

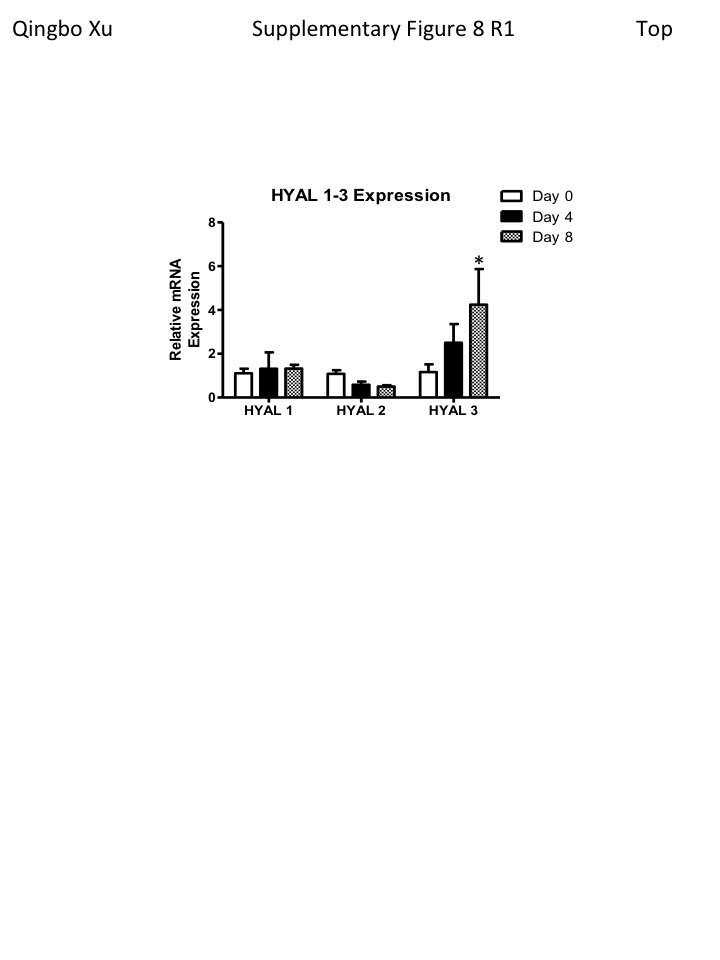

Supplement: Supplementary file 10 — Supplementary Information Figure 8 [file STEM-34-1225-s010.tiff]

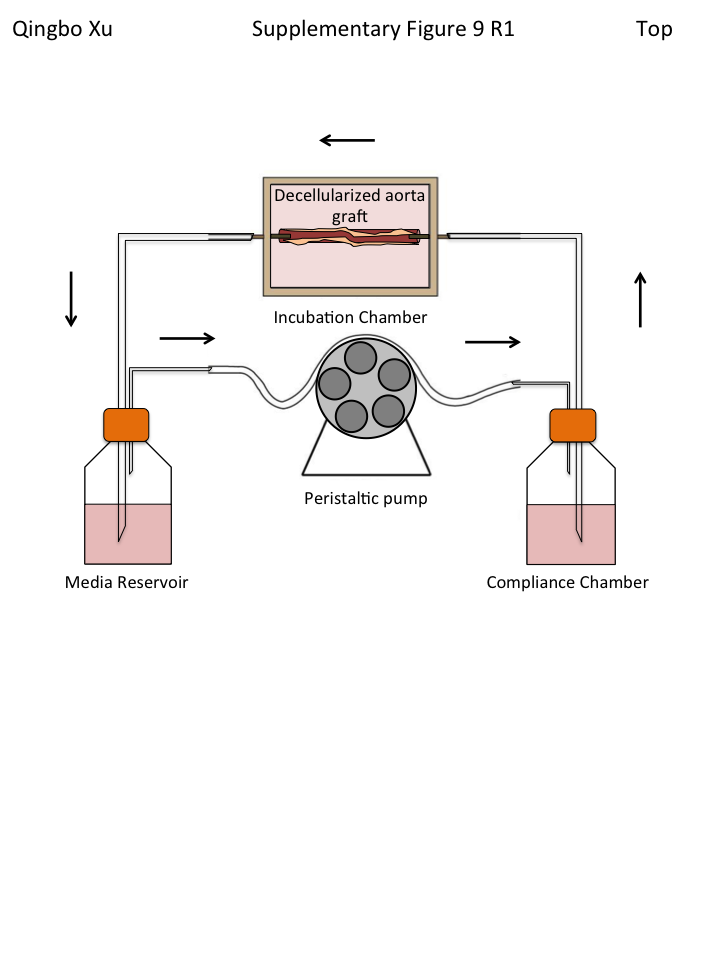

Supplement: Supplementary file 11 — Supplementary Information Figure 9 [file STEM-34-1225-s011.tiff]

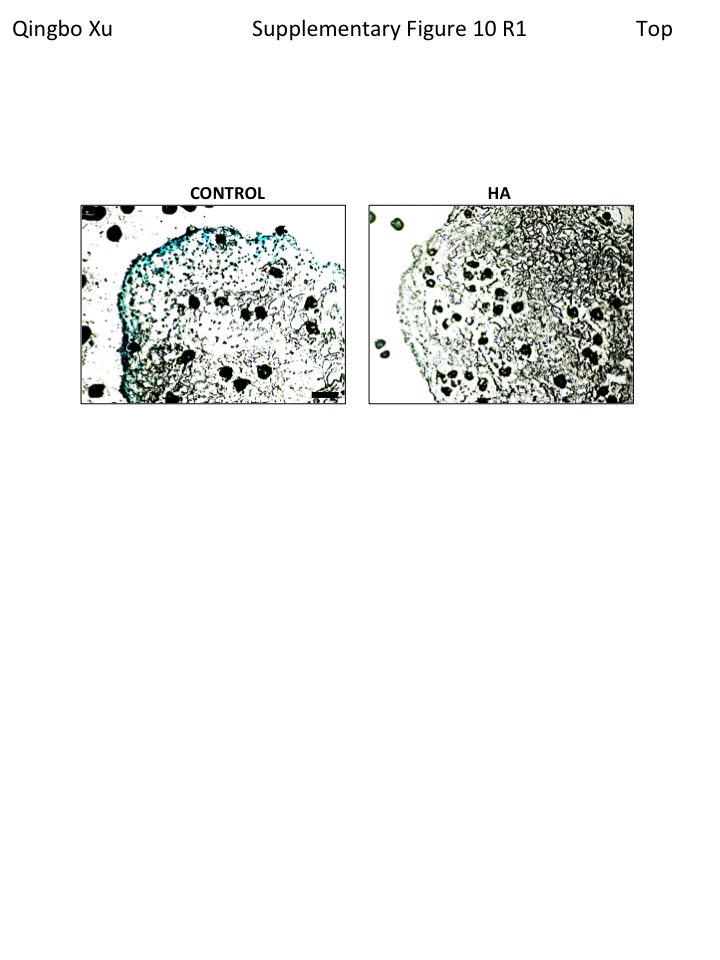

Supplement: Supplementary file 12 — Supplementary Information Figure 10 [file STEM-34-1225-s012.tiff]

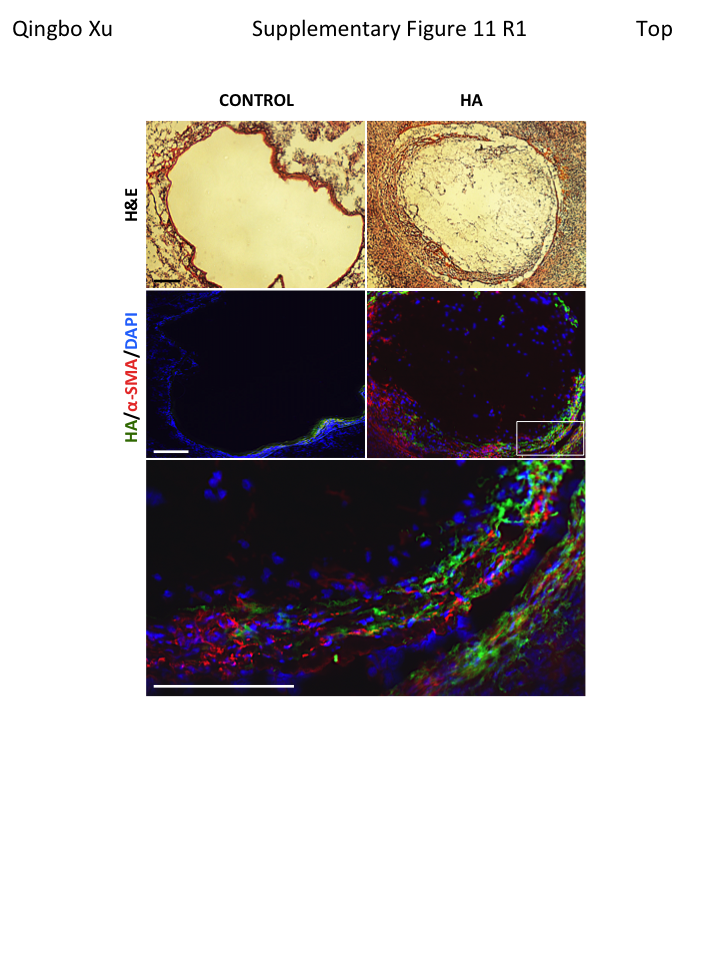

Supplement: Supplementary file 13 — Supplementary Information Figure 11 [file STEM-34-1225-s013.tiff]

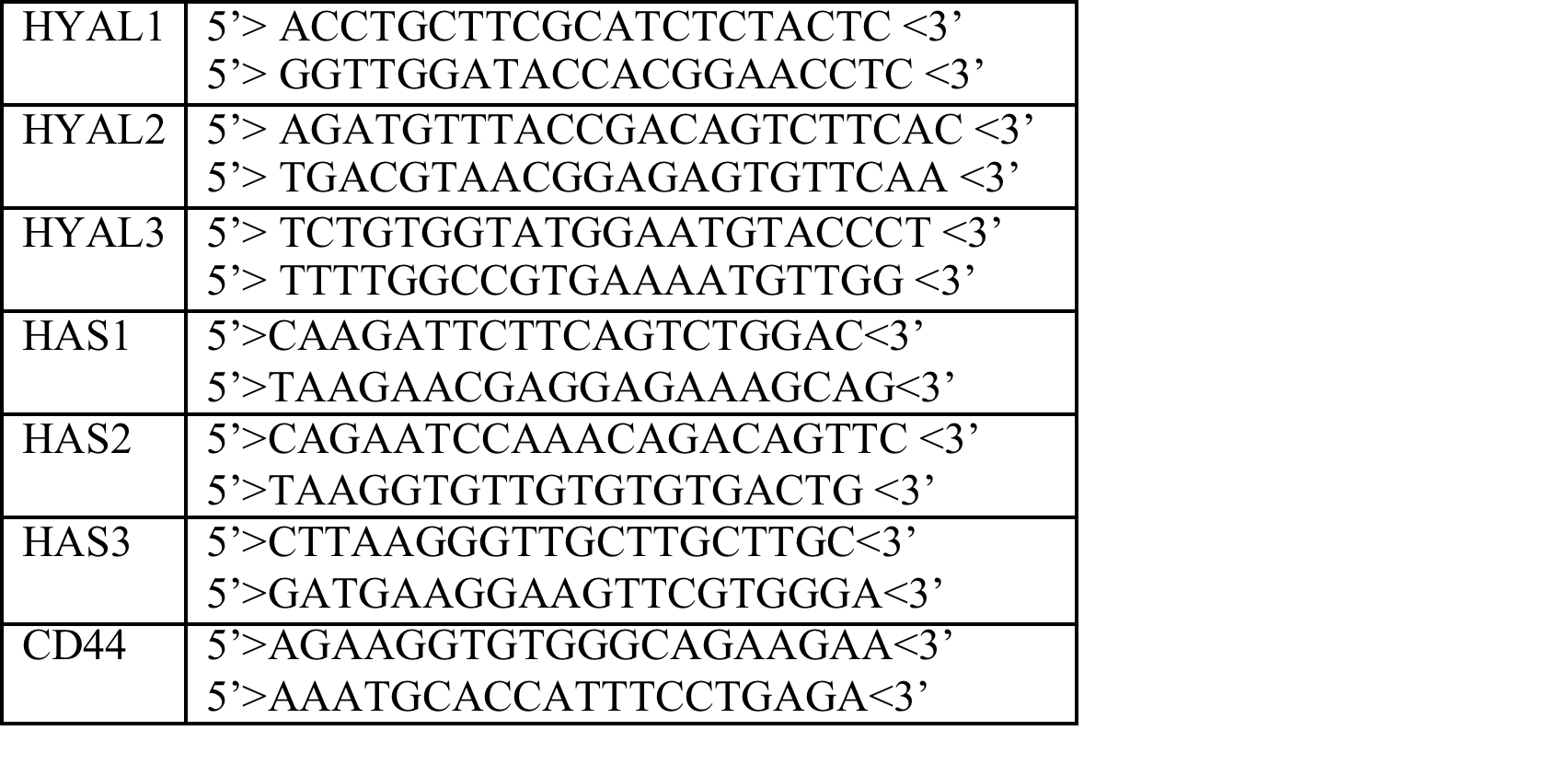


**Supplementary Table S1.** Sequence of forward and reverse primers used in this study

Supplement: Supplementary file 14 — Supplementary Information Table 1 [file STEM-34-1225-s014.docx]
