# Supplementary material for: Hyaluronan Is Crucial for Stem Cell Differentiation into Smooth Muscle Lineage
Source: Stem Cells. 2016 Mar 4;34(5):1225–38. doi: 10.1002/stem.2328 (PMC4864761; doi:10.1002/stem.2328)
Supplement: Supplementary file 2 — Supplementary Information 2 [file STEM-34-1225-s002.docx]

**Supplemental Data**

**Detailed Methods**

**Materials**

HMW-HA (>950 kDa) (GLR002), LMW-HA (15-40 kDa) (GLR001), and neutralizing anti-CD44 antibody were obtained from R&D Systems. PD98059 was purchased from Calbiochem. AG1478 was from Life Technologies. Hyaluronidase (H4272), 4-Methylumbelliferone (4MU) **(**M1381), RGD peptides G4391 (antagonist of integrin function) and S3771 (negative control for G4391), were from Sigma.

**ESC culture and SMC/EC differentiation**

Detailed protocols for mouse ES cell (ES-D3 cell line, CRL-1934; ATCC, Manassas, VA) culture and SMC differentiation were described in our previous studies [1, 2]. Briefly, ESCs were cultured in gelatin-coated flasks in Dulbecco's modified essential medium (DMEM) (ATCC) supplemented with 10% fetal bovine serum (FBS) (ATCC), 10 ng/ml LIF (Chemicon), 0.1 mM 2-mercaptoethanol, 100 U/ml penicillin, 100 μg/ml streptomycin in a humidified incubator supplemented with 5% CO_2_, split at a 1:6 ratio every other day. Cell passages 3-20 were used in this study. Briefly, for SMC differentiation, undifferentiated ES cells were seeded on mouse collagen IV (5 μg/ml)-coated flasks or plates in differentiation medium [DM, MEM α-medium (GIBCO) supplemented with 10% FBS, 0.1 mM 2-mercaptoethanol, 100 U/ml penicillin, and 100 μg/ml streptomycin] for 1-8 days before further treatment. The medium was refreshed every other day. Detailed protocols for EC differentiation were described in our previous studies [3]. Briefly, ESCs were cultured in the exact manner described for SMC differentiation (above) with the addition of 10 ng/ml mouse VEGF-165 (Bender Medsystems) added to the differentiation medium. Mature SMCs were isolated from mouse aorta as described previously [4] and maintained in the SMC specific media SmGM-2 BulletKit (Cat No. CC-3181 & CC-4149), split at a 1:3 ratio every 3 days.

**Cell vitality assays**

Assays were performed using Vitality (VB-48) protocal on a Nucleocounter NC-3000 (ChemoMetec). Experiments were performed according to the manufacturers specifications

**Cell Contractility Assays**

ESC-derived SMCs (esSMCs) were washed with PBS, stimulated with 60 mMKCl (Sigma-Aldrich) in DM, and monitored under the microscope up to 45 min. Movies of the same field were recorded with time-lapse phase contrast microscopy.

**Collagen gel contraction assay**

Collagen I was made up in medium at a final concentration of 150μg/ml. Medium and collagen I were mixed with 5x10^5^ cells and immediately dispensed into 35-mm culture plates and allowed to polymerize at 37^ο^C for 30 min. Immediately after polymerization, 1 ml of culture medium was added to each plate. After incubation for 48 h, the collagen gels were photographed and the area of the gel surface was measured.

**Real-time RT-PCR**

The procedure used for RT-qPCR was similar to that we have described elsewhere [5]. Total RNA was isolated from cells using an RNeasy Mini kit (QIAGEN Inc.) according to manufacturer’s protocol. Briefly, 2 μg RNA were reverse transcribed into cDNA with random primers by MMLV reverse transcriptase (RT) (Promega) and real time RT-PCR was performed using 2ng of cDNA per sample with a SYBR Green Master Mix in a 25-μl reaction. Ct values were measured using ABI PRISM 7000 Sequence Detector (Applied Biosystems) and 18S ribosomal RNA was used as an endogenous control to normalize the amounts of RNA in each sample. Sequences of SMC primer sets used were as previously described by our laboratory [1]. Sequences of other primer sets used in this study are shown in Supplementary Table S1.

**Western Blot Analysis**

Harvested cells were lysed with IP-A buffer (25 mM Tris-HCl pH 7.5, 150 mM NaCl, 1 mM EDTA pH 8.0, 1%Triton X-100 plus protease inhibitors) and proteins were sequentially measured using the Bradford method. 30 μg of lysate was applied to SDS- PAGE before being transferred to a nitrocellulose membrane (Amersham Biosciences), followed by a standard western blotting procedure. Antibodies against α-SMA, Calponin, SM-22α, SMMHC, (all purchased from Abcam), p-EGFR (Tyr1174), EGFR (1005) p-ERK1/2 (Thr-202/Tyr-204) and HAS2 (Y-14) (all purchased from Santa Cruz Biotechnology), were used to detect the respective proteins. Bound primary antibodies were detected using an HRP-conjugated secondary antibody and an ECL detection system (Amersham Biosciences). To confirm equal loading, membranes were stained with antibody against GAPDH (Santa Cruz Biotechnology).

**Immunofluorescence Staining** Cultured cells were fixed with 4% paraformaldehyde (PFA) permeabilised with 0.1% Triton X-100 in PBS and blocked with 10% normal swine serum (Dako). The primary antibodies for OCT4, SOX2, α-SMA, Calponin, SM-22α Smmhc and CD31 were all purchased from Abcam and detected with appropriate secondary Alexafluor 488, 568 or 647 antibodies. Cells were counterstained with 4’,6-diamidino-2-phenylindole (DAPI) or Hoechst 33342 (life technologies) to stain the nuclei and observed using an Axio Imager.M2 microscope and AxioVision Digital Imaging System (Carl Zeiss Ltd.).

**FACS Analysis**

To detect CD44 on cell surface, the trypsinized cells (1x10^6^) were harvested, washed with PBS containing 5% BSA and then fixed in PBS containing 3% formaldehyde. After removal of fixing solution, cells were incubated with rat anti-CD44 antibody or an isotype-matched control antibody, then incubated with a fluorescein isothiocyanate-conjugated secondary antibody, washed and analysed with a FACS scan flow cytometer (Becton Dickinson Immunocytometry Systems, Mountain View, CA, USA).

**Measurement of HA secretion**

HA released into the culture medium was measured using an HABP-based commercial kit according to the manufactures instruction. (Hyaluronic Acid quantitative test kit, from Corgenix). Supernatants were either pooled for global HA concentration at time points throughout differentiation or were taken 24hrs after medium was replenished to measure *de-novo* HA synthesis at specific time points. The quantity of HA secreted was calculated as the ratio of HA in the medium normalized to cell numbers.

**Staining of intracellular and pericellular HA**

To visualize intracellular HA, Cells were fixed with 4% paraformaldehyde (PFA) and permeabilised with 0.1% Triton X-100 in PBS, and stained overnight with biotinylated HA-binding protein (2μg/ml, bHABP, Amsbio,). To visualize pericellular HA, live cells were incubated with bHABP (2μg/ml, bHABP, Amsbio,) for 1h at RT then Incubated with Streptavidin, Alexa Fluor® 488 Conjugate (Life Technologies) for 1h at RT. Fixed Cells were counterstained with 4’,6-diamidino-2-phenylindole (DAPI) or Hoechst 33342 (life technologies) for live cells before image acquisition using the Axio Imager.M2 microscope and AxioVision Digital Imaging System (Carl Zeiss Ltd.).

**Visualization of pericelluar HA by particle exclusion assay**

The exclusion of horse erythrocytes was used as previously described [6] to visualize the HA pericellular coat. Briefly, formalized horse erythrocytes (HB042, TCS, Biosciences) were added to 35-mm dish containing subconfluent cells and swirled gently for even distribution. The dishes were incubated at 37°C for 15 minutes to allow the erythrocytes to settle around the cells. On settling, the erythrocytes are excluded from zones around the cells with HA pericellular coats. These zones are viewed under the microscope as areas of erythrocyte exclusion. Zones of exclusion were visualized on a Zeiss Axiovert 135 inverted microscope.

**Luciferase Reporter Assay**

ESCs were seeded on gelatin-coated 12-well plate prior to transfection with pGL3-SM-22α promoter reporter gene (0.33μg/well) using a Fugene-6-Reagent (Roche Molecular Biochemicals) according to the manufacturer’s instructions. Renilla luciferase (0.1μg/well) was used in all transfection assays as an internal control. Media (+/- 4MU) change was carried out on cells after the overnight transfection. Luciferase and Renilla activities were detected after 48 hours using a standard protocol and relative luciferase unit (RLU) was defined as the ratio of Firefly versus Renilla with that of the control (set as 1.0).

**HAS2 and CD44 siRNA experiments**

HAS2 siRNA (sc-45329) was purchased from Santa Cruz, *Silencer*^®^ Select Negative Control #1 siRNA (43908430) CD44 Silencer® Select Pre-designed siRNA (4390771) were purchased from Life Technologies**.** ESCs were transfected with 20 μl (per 10^6^ cells) of 10μM siRNA using siIMPORTER transfection reagents (Millipore) according to the manufacturer’s protocol provided. Following 48hrs transfection cells were differentiated then harvested after 72 hours and real-time RT-PCR and Western blot analysis were performed.

**Generation of HAS2 adenovirus**

A recombinant HAS2-HA (Mouse) Adenovirus (Cat. No 180863A) and a Null Adenovirus (Cat. No. 000048A) were received from abm and both amplified according to the protocol provided by the company. HEK293 cells were seeded on a 60-70% confluence on a 100mm dish and 200ul of the adenovirus stock were added to the medium. When 95% of the cells had detached, both cells and supernatants were harvested in a 50ml falcon tube. The collection was then subjected to 3 cycles of freeze-thaw in order to release the virus from the cells and then centrifuged for 10min at RT at 3000 rpm. The supernatant collected contained the amplified adenovirus, the titration of which was estimated by using an ELISA QuickTiter™ Adenovirus Titer ELISA Kit from Cambridge bioscience (Cat. No. VPK-110) at 1.3x10^6^ ifu/ml for the Null adenovirus and 1.4x10^6^ ifu/ml for the HAS2-HA adenovirus. For adenoviral transduction in ESCs, cells were seeded on Collagen IV for 24h. Null and HAS2-HA adenovirus were then added to the supernatant at an M.O.I of 130 after which the cells were allowed to grow for 72h. The expression of HAS2 as well as SMC markers was then investigated.

**Co-immunoprecipitation**

Cell lysate was harvested from using ice-cold non-denaturing lysis buffer (Thermo Scientific, Rockford, IL). Co-immunoprecipitation (co-IP) was done using the Thermo Scientific Pierce co-IP kit following the manufacturer's protocol. Briefly, rat anti-CD44 antibody (2 μg/ml) (Abcam) was first immobilized for 2 h using AminoLink Plus coupling resin. The resin was then washed and incubated with cell lysate overnight. After incubation, the resin was again washed and protein eluted using elution buffer. Samples were analyzed by Western blotting (as previously described) using anti-EGF-R antibody, and horseradish peroxidase-conjugated secondary antibodies. The specificity of immunoprecipitation was confirmed by negative control reactions performed with the IgG control.

**Vein Graft Procedure**

All procedures were performed according to protocols approved by the Institutional Committee for Use and Care of Laboratory Animals. The procedure used for vein grafts was similar to that described previously [7]. Briefly, 3-month-old ApoE^−/−^ mice (Jackson Laboratories) were anesthetized using a combination of Hypnorm (25 mg/kg; Veta Pharma, UK) and Hypnovel (25 mg/kg; Roche) administered intraperitoneally. The right common carotid artery was mobilized free from the bifurcation at the distal end toward the proximal, cut in the middle, and a cuff placed over the end. The cuff was made from an autoclavable nylon tube 0.63 mm in diameter outside and 0.5 mm inside (Portex Ltd., Hythe-Kent, UK). The artery was turned inside out over the cuff and ligated. The vena cava vein was harvested from an isogenic donor and grafted between the two ends of the carotid artery by sleeving the ends of the vein over the artery cuff and ligating them together with an 8-0 suture. Vigorous pulsation in the vein conduit confirmed successful engraftment. For cell transfer, esSMCs (5x10^5^) were mixed with 1% Matrigel at 4C, applied to the adventitial side to envelope the graft.

**Graft Harvest and Section Preparations**

The grafts were harvested after 2 weeks. The mice were anaesthetised and fixed as describe above. The middle line incision of neck was made and the surround tissue was removed to expose the whole segment of the vessel graft and left common carotid artery. Normal saline perfusion was done from the right common carotid artery, after the left common carotid artery was then cut open. The graft was harvested from the bilateral end of the cuff and frozen in liquid nitrogen immediately. For frozen sections, the graft was embedded in optimal cutting temperature aqueous compound (VWR Scientific, Bridgeport, NJ) in a cryostat (Microm HM 560, Walldorf, Germany) at -20°C. The cross sections were collected, from different regions of the graft.

**Histology and Lesion Measurement**

The grafts were fixed with 4% formaldehyde, processed by routine histology and embedded in paraffin. Sections (5 μm) obtained at the centre of the graft were stained with hematoxylin and eosin (HE) for histological evaluation. The procedure for lesional area measurement is similar to that previously described [8]. Briefly, the lesion was defined as the region between the lumen and the media. Using a transmission microscope (Zeiss, Jena, Germany) sections were scanned, saved and then overlaid with different lines to trace the lumen and media. The lesion area was determined by subtracting the area of the lumen from the area enclosed by the line inside of the media.

**In vivo angiogenesis assay.**

ESCs were differentiated towards SMCs as described above with or without addition of HMW-HA (esSMCs -/+ HMW-HA). ESC-derived ECs (esECs) were generated as previously described [3]. Briefly ESCs were differentiated on collagenIV in the presence of VEGF (50ng/ml) for 8 days. esECs (0.5x10^6^) were mixed with esSMCs -/+ HMW-HA (0.5x10^6^) in 200μl of Matrigel and injected subcutaneously into the back or flank of TIE2-LacZ transgenic mice which express β-gal under the control of the endothelial-specific protein TIE2 promotor [9]. All esSMCS were labelled with Molecular Probes Vybrant Cell Labelling (MP22885) before the *in vivo* angiogenesis assay to distinguish from esECs. Six injections were conducted for each group. one week later, the mice were sacrificed and the plugs were harvested, frozen in liquid nitrogen, and cryosectioned. Samples were fixed with 4% paraformaldehyde in PBS at 4°C overnight, and then HE staining and immunostaning was performed. Images were assessed with Axioplan 2 imaging microscope.

**X-gal staining.**

The procedure for X-gal staining was similar to that described previously [10]. Briefly, sections were incubated at 37°C for 18 h in PBS supplemented with 1 mg/ml X-Gal (Sigma, St Louis, MO), 5 mM potassium ferricyanide, 5 mM potassium ferrocyanide and 2 mM MgCl2. Sections were rinsed with 3% DMSO in PBS and mounted with the endothelium up on a glass slide (2.6x7.5 cm). Counterstaining was performed using Hoechst 33258 (1 μg/ml). Positive cells were enumerated under the microscope.

**Decellularized Vessel Preparations**

Adult male C57Bl/6 mice were purchased from Charles River (Margate, Kent, UK). The mice were sacrificed by cervical dislocation and fixed in a supine position. The middle line incision was made and the lung was lateralized to expose the thoracic part of descending aorta. Perivascular tissue was removed by forceps and intercostal arteries were ligated with bipolar electrocoagulator (SN 54.131; Martin). Then distal end of descending aorta was cut and washed with saline solution via puncture at ascending aorta. The descending aorta was stored at 4°C in phosphate-buffered saline. Aortic segments were thoroughly decellularised using 0.0075% sodium dodecyl sulphate (SDS) (Sigma Aldrich, St. Louis, MO) by continuous flushing of the lumen with a syringe followed by agitation on an orbital shaker for 2 hours at room temperature.

**Ex Vivo Bioreactor**

Using a dissecting microscope (Zeiss, KL1500 LCD, Germany) with 5- to 10-fold amplification, a cuff (0.65 mm in diameter and 1 mm in length) made of autoclavable nylon tube (Portex, London, UK) was passed through the two ends of decellularized vessel and fixed by ligation twice with an 8-0 silk suture. Then, the decellularized vessel was connected to a specially constructed bioreactor (an *ex vivo* circulation system which is driven by a pump). To study the ESC-derived SMCs migrating across the vessel wall *ex vivo*, ESCs (5x10^5^ in 100 μl) were mixed with Matrigel (5 mg/ml; Becton Dickinson Labware, Bedford, Massachusetts, USA) at 4°C, and applied to the adventitia enveloping the decellualarized vessel for 15 minutes static at room temperature. Decellularized vessel was immersed in the culture medium and the ex vivo circulation system was placed in a 5% CO2 incubator at 37°C for 5 days. A constant shear stress at stepwise rates ranging from 10 to 35 dynes/cm2 was applied to the vessels over a period of 48 hours, after which the vessels remained under constant shear stress of 15 dynes/cm2. Subsequently, media containing HMW-HA or no stimulus as controls were infused within a closed circulation in the vessel without physical contact with the outer surface of the vessel to allow migration of cells into the vessel (and changed every 2 days) After 8 days, the vessels were harvested, embedded and frozen in liquid nitrogen prior to hematoxylin and eosin or immunoflourescent staining.

**En face preparation and immunofluoresence staining and frozen section staining**.

The procedure for en face preparation is similar to that described elsewhere [11]. In short, the tissue-engineered vessels or grafts were harvested, and the samples were fixed with acetone and cut open. The vessel segments were mounted and pinned onto rubber with the lumen opened and facing up. After washing with PBS specimens were placed in a humidified chamber and blocked in 5% swine serum in PBS for 30 min at 37°C and incubated with primary antibodies as described in the immunostaining section above. For frozen section staining, serial 5 mym-thick frozen sections were cut from cryopreserved tissue blocks, fixed in a cold 1:1 acetone 10 minutes, and washed with phosphate-buffered saline (PBS) for 20 minutes, and proceed as described above.

**Statistical Analysis**

Data for this study were presented as the mean and standard error of the mean (SEM) of at least three separate experiments. Analysis was performed using Graphpad Prism V.4 (GraphPad Software, San Diego CA) using two-tailed student’s *t*-test and One-Way Analysis of Variance (ANOVA) with appropriate post test. A value of *P* < 0.05 was considered statistically significant.

1. Xiao Q, Zeng L, Zhang Z et al. Stem cell-derived Sca-1+ progenitors differentiate into smooth muscle cells, which is mediated by collagen IV-integrin alpha1/beta1/alphav and PDGF receptor pathways. **American journal of physiology Cell physiology***.* 2007;292:C342-352.

2. Liu WH, Liu JJ, Wu J et al. Novel mechanism of inhibition of dendritic cells maturation by mesenchymal stem cells via interleukin-10 and the JAK1/STAT3 signaling pathway. **PloS one***.* 2013;8:e55487.

3. Zeng L, Xiao Q, Margariti A et al. HDAC3 is crucial in shear- and VEGF-induced stem cell differentiation toward endothelial cells. **The Journal of cell biology***.* 2006;174:1059-1069.

4. Wang Z, Zhu S, Shen M et al. STAT3 is involved in esophageal carcinogenesis through regulation of Oct-1. **Carcinogenesis***.* 2013;34:678-688.

5. Margariti A, Xiao Q, Zampetaki A et al. Splicing of HDAC7 modulates the SRF-myocardin complex during stem-cell differentiation towards smooth muscle cells. **Journal of cell science***.* 2009;122:460-470.

6. Simpson RM, Meran S, Thomas D et al. Age-related changes in pericellular hyaluronan organization leads to impaired dermal fibroblast to myofibroblast differentiation. **The American journal of pathology***.* 2009;175:1915-1928.

7. Zou Y, Dietrich H, Hu Y et al. Mouse model of venous bypass graft arteriosclerosis. **The American journal of pathology***.* 1998;153:1301-1310.

8. Dietrich H, Hu Y, Zou Y et al. Mouse model of transplant arteriosclerosis: role of intercellular adhesion molecule-1. **Arteriosclerosis, thrombosis, and vascular biology***.* 2000;20:343-352.

9. Schlaeger TM, Bartunkova S, Lawitts JA et al. Uniform vascular-endothelial-cell-specific gene expression in both embryonic and adult transgenic mice. **Proceedings of the National Academy of Sciences of the United States of America***.* 1997;94:3058-3063.

10. Hu Y, Baker AH, Zou Y et al. Local gene transfer of tissue inhibitor of metalloproteinase-2 influences vein graft remodeling in a mouse model. **Arteriosclerosis, thrombosis, and vascular biology***.* 2001;21:1275-1280.

11. Zeng L, Zampetaki A, Margariti A et al. Sustained activation of XBP1 splicing leads to endothelial apoptosis and atherosclerosis development in response to disturbed flow. **Proceedings of the National Academy of Sciences of the United States of America***.* 2009;106:8326-8331.

**Supplementary Figure 1. SMC expression and contractility in esSMCs. (A)** Western blot of SMMHC, α-SMA, sm22-α and calponin, in mature SMCs and esSMCs. **(B)** ESCs, esSMCs and mature SMCs were cultured three-dimensionally in collagen I for 48 h. The collagen gels were then photographed after 48h and the collagen gel contractility was assessed by measuring the gel surface area. Graphs are shown as mean ± SEM six independent experiments. Analyses based on One-way ANOVA, followed by Dunnett's Multiple Comparison Test. **^***^** P<0.01, versus ESC.

**Supplementary Figure 2. Effect of HA conditioned medium on ESC-SMC differentiation**. ESCs were seeded on collagen IV-coated plates (5μg/ml) and cultured in DM for up to 9d with or without treatment with HA synthesis inhibitor 4MU (0.5mmol/L) Supernatants were removed daily and replaced with fresh differentiation medium. Supernatants were frozen until needed. New ESCs were seeded on collagen IV-coated plates (5μg/ml) and cultured in the conditioned medium recovered from day 3,5,7 & 9 for 48hrs. RTq-PCR analysis revealed that conditioned medium from day 3 supernatants resulted in significant induction of calponin, SM22-α and SMMHCII gene expression compared to ESC conditioned medium. Day3 supernatants treated with 4MU showed no change, confirming that the increase in SMC expression was HA specific. All analyses based on Student’s t-test. *P<0.05; versus ESC. The experiments were repeated three times with similar results.

**Supplementary Figure 3.** Intracellular HA was visualized by dual immunofluorescence staining with a biotinylated HA-binding probe to localize HA (green) and SM22-α (red). Day 3 differentiated ESCs reveal prominent intracellular HA staining at the colony core compared to the surrounding outgrowth of SM22-α positive cells. Bars 100μm.

**Supplementary Figure 4. Cell viability assay for 4MU treatment.** ESCs were seeded on collagen IV-coated plates (5μg/ml) and cultured in DM for up to 8d with (lower) or without (upper) treatment with HA synthesis inhibitor 4MU (0.5mmol/L). Cells were stained with VB-48, Acridine Orange (AO) and Propidium Iodide (PI) and analysed using the Vitality Assay and a NucleoCounter® NC-3000™. Polygons and markers in the displayed plots were used to show the distribution of living (lower right quadrant), dead (upper right quadrant) and apoptotic cell (lower left quadrant) populations, which is presented graphically. All analyses based on Student’s t-test. NS; not significant. The experiments were repeated six times for each condition (-/+ 4MU).

**Supplementary Figure 5. Removal of HA pericellular coat in SMCs.** Mature SMCs were cultured with or without (Control) HYAL (200μg/ml) for 72 hr before pericellular HA was visualized using a particle-exclusion assay (top panel) or by immunofluorescence staining with a biotinylated HA-binding probe to localize HA (green) and Hoechst (blue) in live cells (bottom panel). **bars, 100μm**.

**Supplementary Figure 6. Densitometry quantification of HYAL and 4MU effect on esSMCs**. Analyses based on One-way ANOVA, followed by Bonferroni’s Multiple Comparison Test. **^*^**P<0.05; **^**^** P<0.01, **^***^** P<0.001 versus esSMC

**Supplementary Figure 7. esSMCs display significant HAS2 induction.** (A) RT-qPCR analysis shows the expression of HAS1/2/3 in ESCs and esSMCs. (B) Immunofluorescence staining of HAS1/2/3 (green) and phalloidin (red) in esSMCs. Bars, 100μm**.** Images are representative of at least three separate experiments. Analyses based on Student’s t-test. NS: not significance; ***P<0.001 versus ESC.

**Supplementary Figure 8. Expression of HYAL during ESC-SMC differentiation.** ESCs were seeded on collagen IV-coated plates (5μg/ml) and cultured in DM for the indicated times. Undifferentiated ESCs (day 0) were grown on gelatin were used as a control. RT-qPCR analysis shows expression of HYAL1/2/3 throughout differentiation timecourse. Over the course of differentiation HYAL3 expression is significantly augmented (mean ± s.e.m., n=3). All analyses based on Student’s t-test. *P<0.05, versus day 0. The experiments were repeated three times with similar results.

**Supplementary Figure 9.** Schematic representation of the decellularized graft bioreactor flow circuit. The decellularized vessel graft is assembled in the incubation chamber. A peristaltic pump is at the upstream of the incubation chamber to provide stable medium perfusion flow. The media reservoir is at the downstream of the incubation chamber. The compliance chamber is to improve the flow regime. The flow direction is indicated by arrows.

**Supplementary Figure 10. X-gal staining.** esSMCs maintained in normal differentiation medium (control) or HMW-HA (HA) were mixed with esECs added to a Matrigel plug before subcutaneous injection into a LacZ mouse. After 1 week the Matrigel plug was removed and sections were stained for X-gal. Bar 100μm. Any blue staining was localized at the plug edge confirming that cell infiltration was predominantly donor- and not host-derived.

**Supplementary Figure 11.** Vena cava segments were surgically removed from C57BL/6 mice under anesthesia and subsequently grafted into carotid arteries of apoE- deficient recipient mice. esSMCs (5x10^5^) were seeded onto the adventitial side to envelop the vein grafts. Grafted tissue fragments were harvested 2 weeks after surgery and stained with hematoxylin and eosin (H&E) and immunofluorecence stained for HA, α-SMA and DAPI. vein segments were seeded with esSMCs that were differentiated in DM in the absence of (control) or presence of HMW-HA (HA). Bars, 100μm. Notably, HA stimulation encourages esSMCs to produce their own HA.

**Supplementary Table S1.** Sequence of forward and reverse primers used in this study

**Graphical Abstract.** esSMCs (5x10^5^) maintained in HMW-HA were mixed with esECs (5x10^5^) then added to a Matrigel plug before subcutaneous injection into a LacZ mouse. After 1 week the Matrigel plug was removed and sections were stained for the EC marker CD31 (green). esSMCs were labelled with Vybrant (red) before implantation in order to be distinguished from esECs. Vybrant stained esSMCs interacted with esECs to form dense and robust tube-like structures. DAPI (blue). bars, 100 μm
